# *MYB64* and *MYB119* Are Required for Cellularization and Differentiation during Female Gametogenesis in *Arabidopsis thaliana*

**DOI:** 10.1371/journal.pgen.1003783

**Published:** 2013-09-19

**Authors:** David S. Rabiger, Gary N. Drews

**Affiliations:** Department of Biology, University of Utah, Salt Lake City, Utah, United States of America; Peking University, China

## Abstract

In angiosperms, the egg cell forms within the multicellular, haploid female gametophyte. Female gametophyte and egg cell development occurs through a unique process in which a haploid spore initially undergoes several rounds of synchronous nuclear divisions without cytokinesis, resulting in a single cell containing multiple nuclei. The developing gametophyte then forms cell walls (cellularization) and the resulting cells differentiate to generate the egg cell and several accessory cells. The switch between free nuclear divisions and cellularization-differentiation occurs during developmental stage FG5 in *Arabidopsis*, and we refer to it as the FG5 transition. The molecular regulators that initiate the FG5 transition during female gametophyte development are unknown. In this study, we show using mutant analysis that two closely related MYB transcription factors, MYB64 and MYB119, act redundantly to promote this transition. *MYB64* and *MYB119* are expressed during the FG5 transition, and most *myb64 myb119* double mutant gametophytes fail to initiate the FG5 transition, resulting in uncellularized gametophytes with supernumerary nuclei. Analysis of cell-specific markers in *myb64 myb119* gametophytes that do cellularize suggests that gametophytic polarity and differentiation are also affected. We also show using multiple-mutant analysis that *MYB119* expression is regulated by the histidine kinase CKI1, the primary activator of two-component signaling (TCS) during female gametophyte development. Our data establish a molecular pathway regulating the FG5 transition and implicates CKI1-dependent TCS in the promotion of cellularization, differentiation, and gamete specification during female gametogenesis.

## Author Summary

Female gamete formation in angiosperms occurs through a unique process in which a haploid spore initially undergoes a series of free nuclear divisions without cytokinesis, resulting in a single cell containing multiple nuclei. The nuclei then differentiate and are partitioned with cell walls to generate the egg cell and several accessory cells. The molecular regulators that initiate the switch between free nuclear divisions and differentiation during female gametophyte development are unknown. In this study we show that two transcription factors, MYB64 and MYB119, redundantly act to promote this process in the model organism *Arabidopsis*. We also show that one of them, *MYB119*, is transcriptionally regulated by the histidine-kinase CKI1. Our data establish the framework of a gene regulatory network required to promote cellularization, differentiation, and gamete specification during female gametogenesis.

## Introduction

The alternation between haploid gametophyte and diploid sporophyte generations is a fundamental aspect of the plant life cycle. In all species, gametophytes are essential for gamete formation. Angiosperms have two gametophytes: a female gametophyte, which is also referred to as the embryo sac, and a male gametophyte, which is also called the pollen grain. In angiosperms, the egg and sperm cells form within the female and male gametophytes, respectively.

The angiosperm female gametophyte most commonly consists of one egg cell, one central cell, two synergid cells, and three antipodal cells, and the male gametophyte contains two sperm cells encased within a vegetative cell. The female and male gametophytes develop within the flower's sexual organs and are spatially separated. During sexual reproduction, the male gametophyte forms a pollen tube that grows through the floral tissues to deliver its two sperm cells to the female gametophyte. Following sperm cell delivery, the egg cell and central cell both become fertilized and subsequently give rise to the embryo and endosperm of the seed, respectively. The synergid cells are required to attract the pollen tube. However, the function of the three antipodal cells is currently unknown [1], [2].

**Figure 1:**
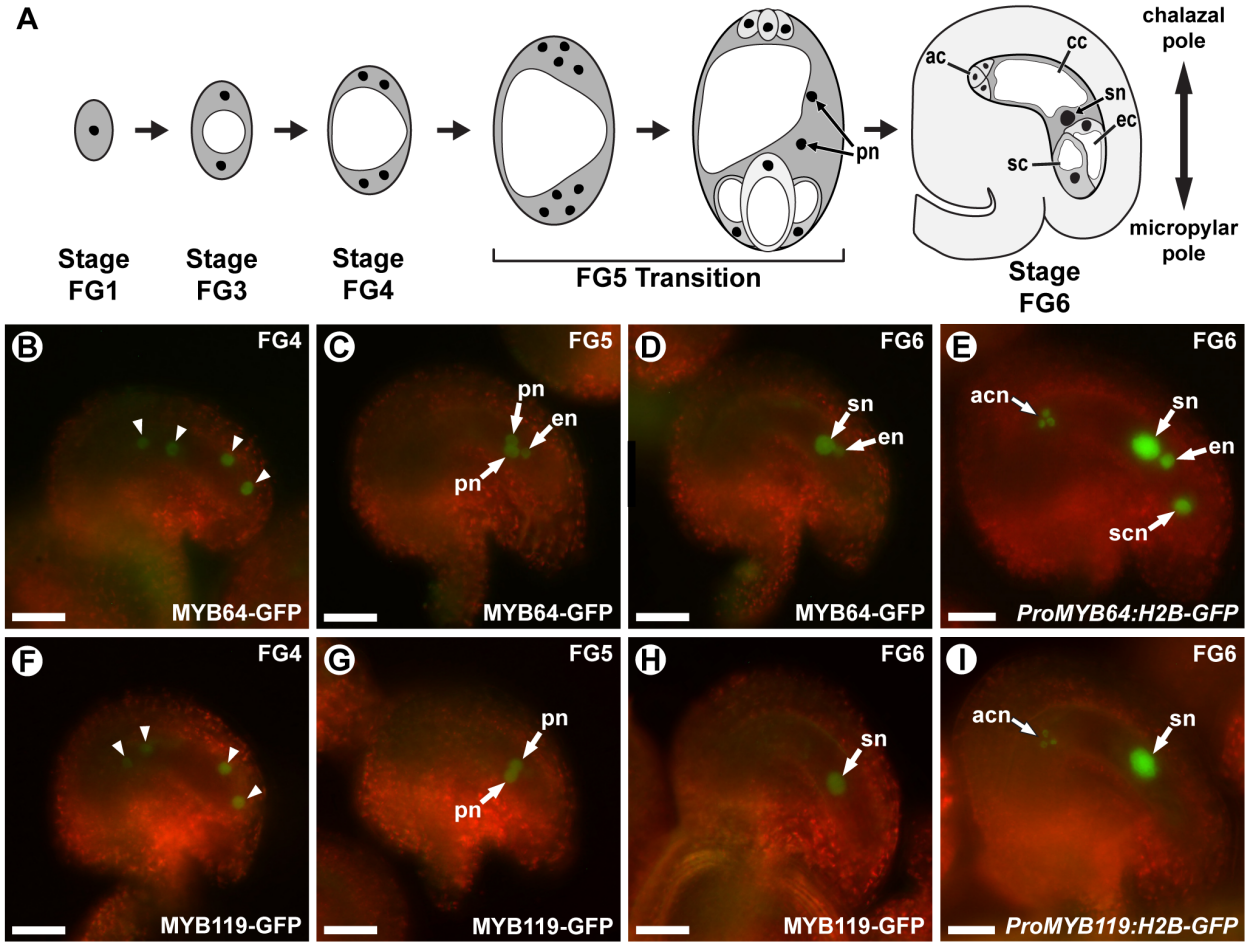
*MYB64* and *MYB119* are expressed during female gametogenesis. (A) A schematic of female gametogenesis in *Arabidopsis thaliana*. Following meiosis, a haploid spore (FG1) undergoes two rounds of synchronous nuclear divisions without cytokinesis to generate a four-nucleate coenocyte (FG4). A third division is immediately followed by cellularization and differentiation (FG5 Transition) to generate the seven cells of the female gametophyte. Two polar nuclei in the central cell then fuse to form the secondary nucleus of the central cell (FG6). ac, antipodal cell; cc, central cell; ec, egg cell; pn, polar nuclei of the central cell; sc, synergid cell; sn, secondary nucleus (fused polar nuclei of the central cell). (B–D and F–H) Epifluorescent micrographs of wild-type female gametophytes expressing *ProMYB64:MYB64-GFP* (B–D), or *ProMYB119:MYB119-GFP* (F–H). (B, F) Expression at stage FG4. GFP is observed in all four nuclei (arrowheads) for both constructs. (C, G) Expression at stage FG5. GFP is observed in the unfused polar nuclei for both constructs. MYB64-GFP is additionally observed in the egg cell nucleus. (D, H) Expression at stage FG6. GFP is observed in the secondary nucleus for both constructs. MYB64-GFP is additionally observed in the egg cell nucleus. (E and I) Epifluorescent micrographs of wild-type female gametophytes expressing *ProMYB64:H2B-GFP* (E) and *ProMYB119:H2B-GFP* (I). For *ProMYB64:H2B-GFP*, GFP is observed in all nuclei of the female gametophyte (E). For *ProMYB119:H2B-GFP*, GFP is observed in the nuclei of the antipodal cells and central cell (I). acn, antipodal cell nuclei; en, egg nucleus; pn, unfused polar nuclei of the central cell; scn, synergid cell nucleus; sn, secondary nucleus (fused polar nuclei of the central cell). Scale bars are 25 µm.

Female gamete specification occurs during female gametophyte development, also referred to as female gametogenesis. During female gametogenesis (Figure 1A), the developing embryo sac initially goes through a coenocytic phase, during which a haploid megaspore undergoes two rounds of mitosis without cytokinesis (stages FG1–FG4). These nuclear divisions are accompanied by rapid cell growth resulting in an enlarged four-nucleate coenocyte. Gametogenesis then undergoes a major developmental transition: the coenocytic developmental pattern ceases, and during a third round of mitosis, division is accompanied by phragmoplast and cell plate formation, resulting in the nuclei becoming surrounded by cell walls (cellularization). In addition to cellularization, mitosis ceases, cell growth attenuates, and the resulting cells differentiate [1], [3], [4]. All of these post-coenocytic events occur during stage FG5, we therefore refer to this transition as the FG5 transition (Figure 1A).

The molecular pathways that regulate the FG5 transition are not understood. This transition involves the regulation of multiple processes including cell wall formation, cell-cycle regulation, cell growth, and cellular differentiation. Regulatory genes that control all of these processes have not been identified. However, a few mutants affected in a subset of these processes have been characterized, including *retinoblastoma related* (*rbr*) and *cytokinin independent 1* (*cki1*).

*RBR* encodes a homolog of the tumor suppressor gene pRb and performs an evolutionarily conserved role in *Arabidopsis* to suppress entry into S-phase of the cell cycle. Mutations affecting *RBR* result in additional nuclear divisions during female gametogenesis. The extra divisions most often occur post-cellularization, resulting in cells with supernumerary nuclei, but occasionally occur prior to cellularization, resulting in the production of extra egg or synergid cells [5]–[9]. Additionally, *rbr* gametophytes fail to express some cell specific markers, suggesting that they are defective in cell differentiation [9].

*CKI1* encodes an *Arabidopsis* histidine kinase (AHK) related to the three cytokinin receptors AHK2, AHK3, and AHK4 [10]. In contrast to AHK2–AHK4, the extracellular domain of CKI1 does not bind cytokinins [11]. However, ectopic expression of *CKI1* induces constitutive cytokinin-like responses in the absence of cytokinin, and this activity involves the downstream components of the cytokinin two-component signaling (TCS) pathway [10], [12]–[15]. *cki1* mutants are defective in female gametogenesis starting at stage FG5, have cellularization defects, and occasionally contain supernumerary nuclei [16]–[18]. Mutations affecting TCS components also affect the female gametophyte and exhibit phenotypes similar to *cki1* [18], [19]. By contrast, analysis of *ahk2 ahk3 ahk4* triple mutants indicates that AHK2–AHK4 are not necessary for female gametophyte development [18]–[23]. These observations suggest that CKI1 activates the TCS pathway independent of cytokinin within the female gametophyte. However, it is unclear what developmental processes the CKI1-dependent TCS pathway regulates during female gametogenesis.

Here, we show that *MYB64* and *MYB119* act redundantly to promote all aspects of the FG5 transition during female gametogenesis in *Arabidopsis*. *MYB64* and *MYB119* are predicted to encode two closely related R2R3-MYB transcription factors that are expressed during the FG5 transition. We also show that *MYB119* is regulated by CKI1, providing new insights into the molecular functions of CKI1 within the female gametophyte.

## Results

### *MYB64* and *MYB119* are expressed during female gametogenesis and encode nuclear-localized proteins

We previously identified *MYB64* (*At5g11050*) and *MYB119* (*At5g58850*) in a differential expression screen for transcription factor genes expressed in the female gametophyte [24]. In that study, expression in the mature female gametophyte was verified for *MYB64*, but not *MYB119*, using a transcriptional reporter. To confirm the expression patterns of *MYB64* and *MYB119* and to characterize the expression of these genes throughout female gametophyte development, we analyzed transgenic plants containing translational GFP fusion constructs (*ProMYB64:MYB64-GFP* and *ProMYB119:MYB119-GFP*). *ProMYB64:MYB64-GFP* and *ProMYB119:MYB119-GFP* individually were capable of complementing the seed phenotype of *myb64 myb119* double mutants discussed below (Table S1). Both fusion proteins were localized to nuclei, which is consistent with their predicted role in transcriptional regulation (Figure 1B–1D and 1F–1H).

*ProMYB64:MYB64-GFP* and *ProMYB119:MYB119-GFP* expression was transient during female gametogenesis. Both fusion proteins were first detected in the coenocytic female gametophyte at stage FG4 (four-nucleate stage). At this stage, MYB64-GFP and MYB119-GFP were detected in all four nuclei of the female gametophyte (Figure 1B and 1F). Post-cellularization, both fusion proteins were detected in the central cell (unfused polar nuclei at stage FG5 and secondary nucleus at stage FG6) (Figure 1C, 1D, 1G and 1H). MYB64-GFP was additionally detected in the egg cell nucleus during stages FG5 and FG6 (Figure 1C and 1D). The levels of both fusion proteins were dramatically reduced in mature female gametophytes (stage FG7): MYB119-GFP was not detectable, while MYB64-GFP expression was very weak and detectable in only a minority (26%) of gametophytes.

We also generated and analyzed transcriptional fusions for both *MYB64* and *MYB119* (*ProMYB64:H2B-GFP* and *ProMYB119:H2B-GFP*). The transcriptional fusions had expanded, but overlapping expression patterns relative to their respective translational fusions: *ProMYB64:H2B-GFP* expression was detected in all cells of the female gametophyte and *ProMYB119:H2B-GFP* expression was detected in the antipodal cells in addition to the central cell (Figure 1E and 1I). In addition to the female gametophyte, *ProMYB64:H2B-GFP* and *ProMYB119:H2B-GFP* expression was also detected in the septum of the ovary (Figure S1A and S1B).

**Figure 2:**
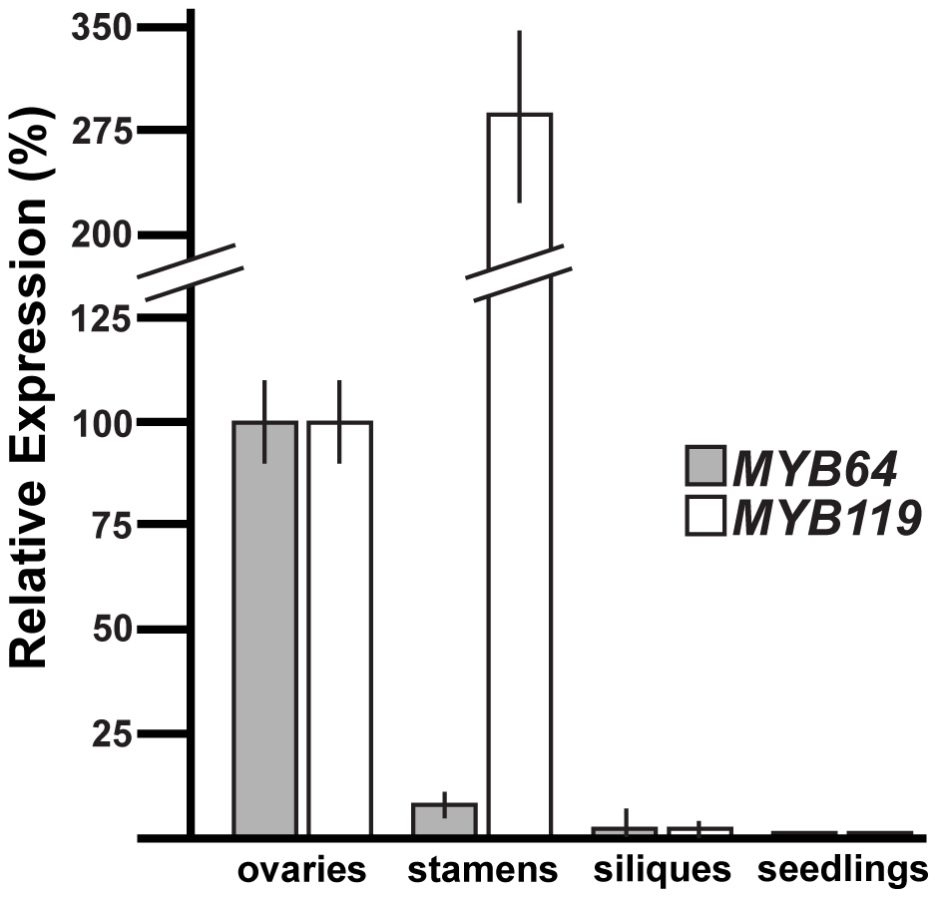
qRT-PCR analysis of *MYB64* and *MYB119* expression. Relative expression of *MYB64* and *MYB119* in pistils, stamens, siliques at 36 hours after pollination, and seedlings at 10 days after germination. Error bars indicate standard deviations.

To determine whether *MYB64* and *MYB119* are expressed elsewhere in the plant, we used quantitative real-time PCR (qRT-PCR) with cDNA from a variety of plant tissues (Figure 2). Consistent with the female gametophyte expression of *ProMYB64:MYB64-GFP* and *ProMYB119:MYB119-GFP*, strong expression was detected for both genes in the ovary, which contains the female gametophyte. Little to no expression was detected in siliques at 36 hours after pollination, and expression was not detected for either gene in 10-day-old seedlings consisting of roots, stems and leaves. By contrast, strong expression of *MYB119* was detected in stamens. To localize expression within stamens, we analyzed *GFP* expression of *ProMYB64:H2B-GFP* and *ProMYB119:H2B-GFP* in male reproductive tissue. We did not detect any GFP expression in the male gametophyte for either construct; however, strong GFP expression was detected in the filament for *ProMYB119:H2B-GFP* (Figure S1C).

In summary, *MYB64* and *MYB119* encode nuclear-localized proteins and are expressed within the female gametophyte. Furthermore, these genes are expressed during a specific period of female gametogenesis, from just before the FG5 transition through stage FG6.

### Transmission of the *myb64 myb119* double mutation through the female gametophyte is affected

To determine whether *MYB64* and *MYB119* are required for female gametophyte development, we obtained T-DNA insertion mutants for both genes from the *Arabidopsis* Biological Resource Center (ABRC) [25]–[27]. We analyzed two alleles of *MYB64* (*myb64-1* and *myb64-4*) and three alleles of *MYB119* (*myb119-1*, *myb119-3*, and *myb119-5*) (Figure S2) (see Table S2 for additional alleles not discussed in this paper). With all five single mutants, defects in vegetative or reproductive tissues were not apparent and siliques contained full seed set. Confocal scanning laser microscopy (CSLM) analysis of *myb64-1* and *myb119-3* ovules indicated that female gametophyte development was unaffected in these mutants (Figure S3).

The absence of mutant phenotypes in *myb64* and *myb119* single mutants, together with the overlapping expression patterns and high sequence similarity of these two genes [28], suggested that *MYB64* and *MYB119* may be functionally redundant in the female gametophyte. To test this, we analyzed transmission of multiple *myb64 myb119* mutant allele combinations (Table 1 and Table 2). In all mutant allele combinations tested, self-fertilized *myb64/MYB64 myb119/myb119* plants segregated ∼1∶1 for *myb64/MYB64* and *MYB64/MYB64* progeny (Table 1). Similarly, self-fertilized *myb64/myb64 myb119/MYB119* plants segregated ∼1∶1 for *myb119/MYB119* and *MYB119/MYB119* progeny (Table 2). These results suggest that gametophytic transmission of the *myb64 myb119* double mutation is affected.

**Table 1:** Segregation of *myb64* alleles.

Parental Genotype		Segregation of *myb64* Allele in Progeny			
Female	Male	+/+	−/+	−/−	p-val
*myb64-1/MYB64 myb119-3/myb119-3*	*myb64-1/MYB64 myb119-3/myb119-3*	55	53	0	6.7E-13^a^
*myb64-1/MYB64 myb119-3/myb119-3*	*MYB64/MYB64 MYB119/MYB119*	122	0	N/A	<2.2E-16^b^
*MYB64/MYB64 MYB119/MYB119*	*myb64-1/MYB64 myb119-3/myb119-3*	74	69	N/A	0.68^b^
*myb64-4/MYB64 myb119-1/myb119-1*	*myb64-4/MYB64 myb119-1/myb119-1*	51	55	1	6.8E-11^a^
*myb64-4/MYB64 myb119-1/myb119-1*	*MYB64/MYB64 MYB119/MYB119*	113	2	N/A	<2.2E-16^b^
*MYB64/MYB64 MYB119/MYB119*	*myb64-4/MYB64 myb119-1/myb119-1*	72	63	N/A	0.4386^b^

a *X* ^2^ test for an expected segregation ratio of 1∶2∶1.

b *X* ^2^ test for an expected segregation ratio of 1∶1.

+, wild-type allele; −, mutant allele; N/A, not applicable.

**Table 2:** Segregation of *myb119* alleles.

Parental Genotype		Segregation of *myb119* Allele in Progeny			
Female	Male	+/+	−/+	−/−	p-val
*myb64-1/myb64-1 myb119-3/MYB119*	*myb64-1/myb64-1 myb119-3/MYB119*	44	39	0	6.4E-11^a^
*myb64-1/myb64-1 myb119-3/MYB119*	*MYB64/MYB64 MYB119/MYB119*	133	0	N/A	<2.2E-16^b^
*MYB64/MYB64 MYB119/MYB119*	*myb64-1/myb64-1 myb119-3/MYB119*	98	90	N/A	0.56^b^
*myb64-1/myb64-1 myb119-5/MYB119*	*myb64-1/myb64-1 myb119-5/MYB119*	496^c^	519^c^	0^c^	<2.2E-16^a^
*myb64-1/myb64-1 myb119-5/MYB119*	*MYB64/MYB64 MYB119/MYB119*	200^c^	0^c^	N/A	<2.2E-16^b^
*MYB64/MYB64 MYB119/MYB119*	*myb64-1/myb64-1 myb119-5/MYB119*	170^c^	172^c^	N/A	0.91^b^

a *X* ^2^ test for an expected segregation ratio of 1∶2∶1.

b *X* ^2^ test for an expected segregation ratio of 1∶1.

c Genotype inferred by glufosinate resistance linked to the *myb119-5* T-DNA.

+, wild-type allele; −, mutant allele; N/A, not applicable.

To determine whether transmission of the *myb64 myb119* double mutation is affected through the female gametophyte and/or male gametophyte, we performed reciprocal crosses of *myb64/myb64 myb119/MYB119* and *myb64/MYB64 myb119/myb119* with wild type. For all allele combinations tested, transmission of *myb64 myb119* double mutations was not significantly affected through the male gametophyte (Table 1 and Table 2). To confirm that male gametophyte development was unaffected, we stained mature pollen grains from *myb64-1/MYB64 myb119-3/myb119-3* plants with DAPI and found that they were phenotypically wild type (*N* = 113) (Figure S4).

In contrast to male gametophyte transmission, transmission of *myb64 myb119* double mutations through the female gametophyte was severely reduced (Table 1 and Table 2). We did not detect any transmission of *myb64-1 myb119-3* and *myb64-1 myb119-5* double mutations through the female gametophyte. However, the *myb64-4 myb119-1* double mutation was transmittable through the female gametophyte at very low frequency (<2%) (Table 1). This partial penetrance allowed us to isolate lines doubly homozygous for *myb64-4* and *myb119-1*. *myb64-4 myb119-1* double-homozygous plants had no obvious vegetative phenotypes.

### *myb64 myb119* female gametophytes fail to exit coenocytic development

**Figure 3:**
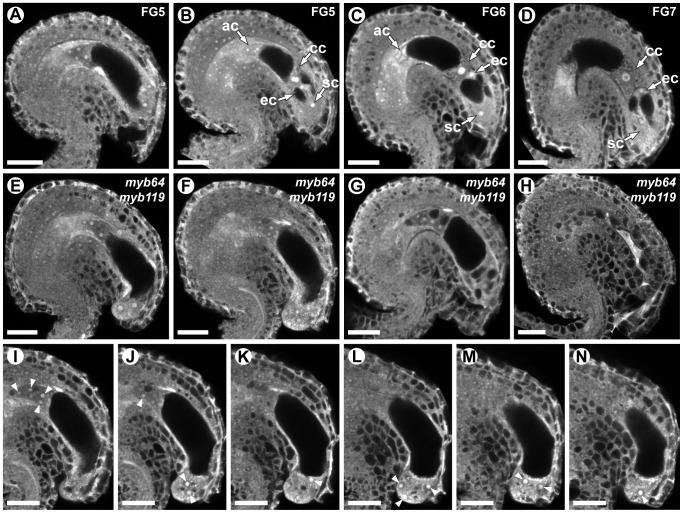
*myb64 myb119* gametophytes fail to initiate the FG5 transition. (A–D) CSLM micrographs of wild-type female gametophytes. (A) A coenocytic, eight-nucleate female gametophyte at stage FG5. Only seven nuclei are visible in this projection. (B) A cellularized eight-nucleate female gametophyte at stage FG5. (C) A wild-type female gametophyte at stage FG6. (D) A mature wild-type female gametophyte at stage FG7. (E–H) CSLM micrographs of *myb64-1 myb119-3* female gametophytes. (E) A coenocytic, eight-nucleate *myb64 myb119* female gametophyte protruding from the micropyle. (F) An enlarged coenocytic *myb64 myb119* female gametophyte containing supernumerary nuclei. (G) A cellularized *myb64 myb119* gametophyte containing supernumerary, atypical cells. (H) A *myb64 myb119* gametophyte that has collapsed and degenerated. (I–N) A Z-stack series of the ovule depicted in F. Fourteen nuclei are indicated by arrowheads. Abbreviations: ac, antipodal cells; cc, central cell; ec, egg cell; sc, synergid cell; WT, wild type. Scale bars are 20 µm.

To determine whether *myb64 myb119* mutations affect female gametophyte development, we analyzed ovules from wild-type and *myb64-1/MYB64 myb119-3/myb119-3* plants using CSLM (Figure 3). During coenocytic development (stages FG1–FG4), *myb64-1 myb119-3* female gametophytes were indistinguishable from wild type. Abnormal *myb64-1 myb119-3* female gametophytes were first apparent beginning at stage FG5, during which wild-type female gametophytes cellularize and differentiate (Figure 3A and 3B). At this time point, *myb64-1 myb119-3* gametophytes had eight nuclei but were not cellularized and were over-expanded, causing the embryo sac to protrude from the micropyle of the ovule (Figure 3E and S5A–S5E). As development progressed, *myb64-1 myb119-3* gametophytes continued to expand and underwent additional nuclear divisions, resulting in enlarged, single-celled gametophytes containing supernumerary nuclei (Figure 3F and 3I–3N). The number of nuclei in these coenocytic gametophytes was variable, ranging from 10 to 18 with an average of 13.5 (+/−2.2) (*N* = 24). At maturity, 46% of the *myb64-1 myb119-3* gametophytes were collapsed and degenerated (Figure 3H), 32% were enlarged multi-nucleate coenocytes, and 22% were cellularized (*N* = 215). Cellularized *myb64 myb119* gametophytes contained extra cells and exhibited little or no morphological similarity to wild-type gametophytes (compare Figures 3C and 3D to Figure 3G and Figure S5F–S5J).

We also analyzed mature ovules of *myb64-4 myb119-1* double-homozygous plants. *myb64-4 myb119-1* female gametophytes had a similar but slightly weaker phenotype relative to that of *myb64-1 myb119-3* discussed above. At maturity, fewer *myb64-4 myb119-1* gametophytes were collapsed and degenerated compared to those from *myb64-1 myb119-3* plants (31% versus 46%, respectively). Additionally, *myb64-4 myb119-1* gametophytes cellularized more frequently than *myb64-1 myb119-3* gametophytes (52% versus 22%, respectively) (*N* = 114).

In summary, most *myb64 myb119* gametophytes fail to cellularize, cease nuclear division, and attenuate cell growth, resulting in enlarged coenocytes with supernumerary nuclei. These data suggest that *MYB64 and MYB119* are required for the FG5 transition during female gametogenesis.

### Cell differentiation and gametophytic polarity are affected in *myb64 myb119* gametophytes

**Figure 4:**
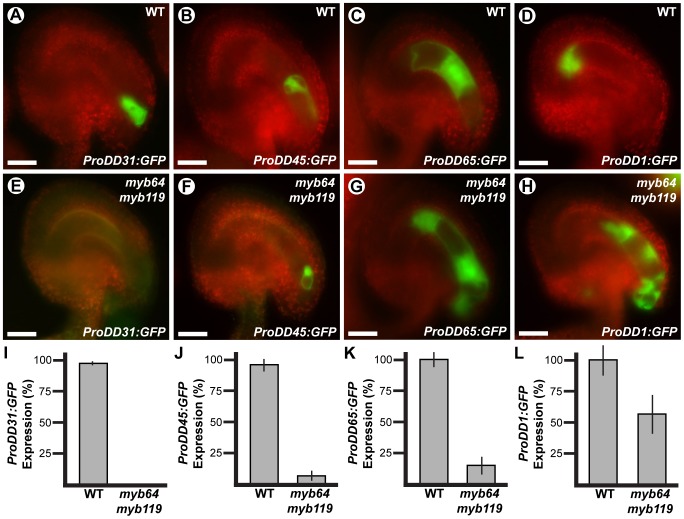
Cell-specific markers are misexpressed in *myb64 myb119* gametophytes. Epifluorescent micrographs of *GFP* expression in wild-type (A–D) and *myb64-1 myb119-3* (E–H) female gametophytes. (A,E) Synergid cell marker *ProDD31:GFP*. (B,F) Egg cell marker *ProDD45:GFP*. (C,G) Central cell marker *ProDD65:GFP*. (D,H) Antipodal cell marker *ProDD1:GFP*. (I–L) Quantification of cell-specific marker expression in gametophytes from *myb64-1/myb64-1 myb119-3/MYB119* plants. Bars represent the percentage of gametophytes expressing GFP. Wild-type expression was calculated as (GFP positive wild type)/(1/2 Total). Mutant expression was calculated as (GFP positive mutant)/(1/2 Total). Error bars indicate standard deviations. WT, wild type. Scale bars are 25 µm.

To determine if cellular differentiation is also affected in *myb64 myb119* gametophytes, we analyzed the expression of several cell-specific GFP markers in *myb64 myb119* gametophytes. The markers analyzed were *ProDD31:GFP*, which is expressed in the synergid cells (Figure 4A); *ProDD45:GFP*, which is expressed in the egg cell (Figure 4B); *ProDD65:GFP*, which is expressed in the central cell (Figure 4C); and *ProDD1:GFP*, which is expressed in the antipodal cells (Figure 4D) [29]. Using crosses, we generated *myb64-1/myb64-1 myb119-3/MYB119* plants homozygous for each respective GFP marker and scored GFP expression in mature female gametophytes from these plants.

The expression of all markers tested was affected in *myb64 myb119* female gametophytes. The synergid cell marker (*ProDD31:GFP*) was the most severely affected and was not detected in *myb64 myb119* gametophytes (Figure 4E and 4I). Expression of the egg and central cell markers was also strongly affected. *ProDD45:GFP* and *ProDD65:GFP* were expressed in 7% and 15% of *myb64 myb119* gametophytes, respectively (Figure 4J and 4K). When expressed, *ProDD45:GFP* was detected in a single, egg-like cell at the micropylar end (Figure 4F), whereas *ProDD65:GFP* was abnormally expressed throughout the female gametophyte (Figure 4G). By contrast, the antipodal cell marker was expressed at a much higher frequency in *myb64 myb119* female gametophytes (57%) (Figure 4L), although it was also abnormally expressed throughout the female gametophyte (Figure 4H).

In summary, *myb64 myb119* female gametophytes either do not express cell-specific markers or express them in an atypical pattern, suggesting that *MYB64* and *MYB119* are required for proper cell differentiation. The expression of micropylar cell markers was either absent or severely reduced, and chalazal cell markers had expanded expression domains; this suggests that gametophytic polarity is also affected in *myb64 myb119* gametophytes, with an expansion of chalazal cell identity at the expense of micropylar cell identity.

### *myb64 myb119* siliques contain autonomous seed-like structures

With all allele combinations tested, siliques from *myb64/MYB64 myb119/myb119* or *myb64/myb64 myb119/MYB119* plants contained ∼50% normal seeds and ∼50% defective seeds (Table 3). Correspondingly, siliques from *myb64-4 myb119-1* double-homozygous plants contained mostly (∼97%) defective seeds (Table 3). In all cases, the defective seeds consisted of mostly desiccated ovules and a smaller proportion of white or collapsed seed-like structures (Figure S6 and Table 3).

**Table 3:** Analysis of *myb64 myb119* seed phenotypes.

	Seed Phenotypic Classes^a^			
Parental Genotype	Desiccated Ovules	Seed-Like Structures	Wild-Type Seeds	*N*
*MYB64/MYB64 MYB119/MYB119*	0.5% (0.7%)	0.4% (0.7%)	99.1% (0.7%)	578
*myb64-1/MYB64 myb119-3/myb119-3*	41.5% (7.7%)	8.5% (5.2%)	50.0% (10.4%)	600
*myb64-1/myb64-1 myb119-5/MYB119*	41.6% (7.5%)	7.3% (5.3%)	51.1% (6.0%)	578
*myb64-4/MYB64 myb119-1/myb119-1*	35.6% (5.4%)	11.6% (4.8%)	52.8% (5.3%)	1536
*myb64-4/myb64-4 myb119-1/myb119-1*	68.5% (7.0%)	28.2% (5.6%)	3.3% (2.7%)	619

a Plants of the indicated genotypes were allowed to self-pollinate and the phenotypes of the resulting seeds were scored. Percentages in parentheses are standard deviations.

**Figure 5:**
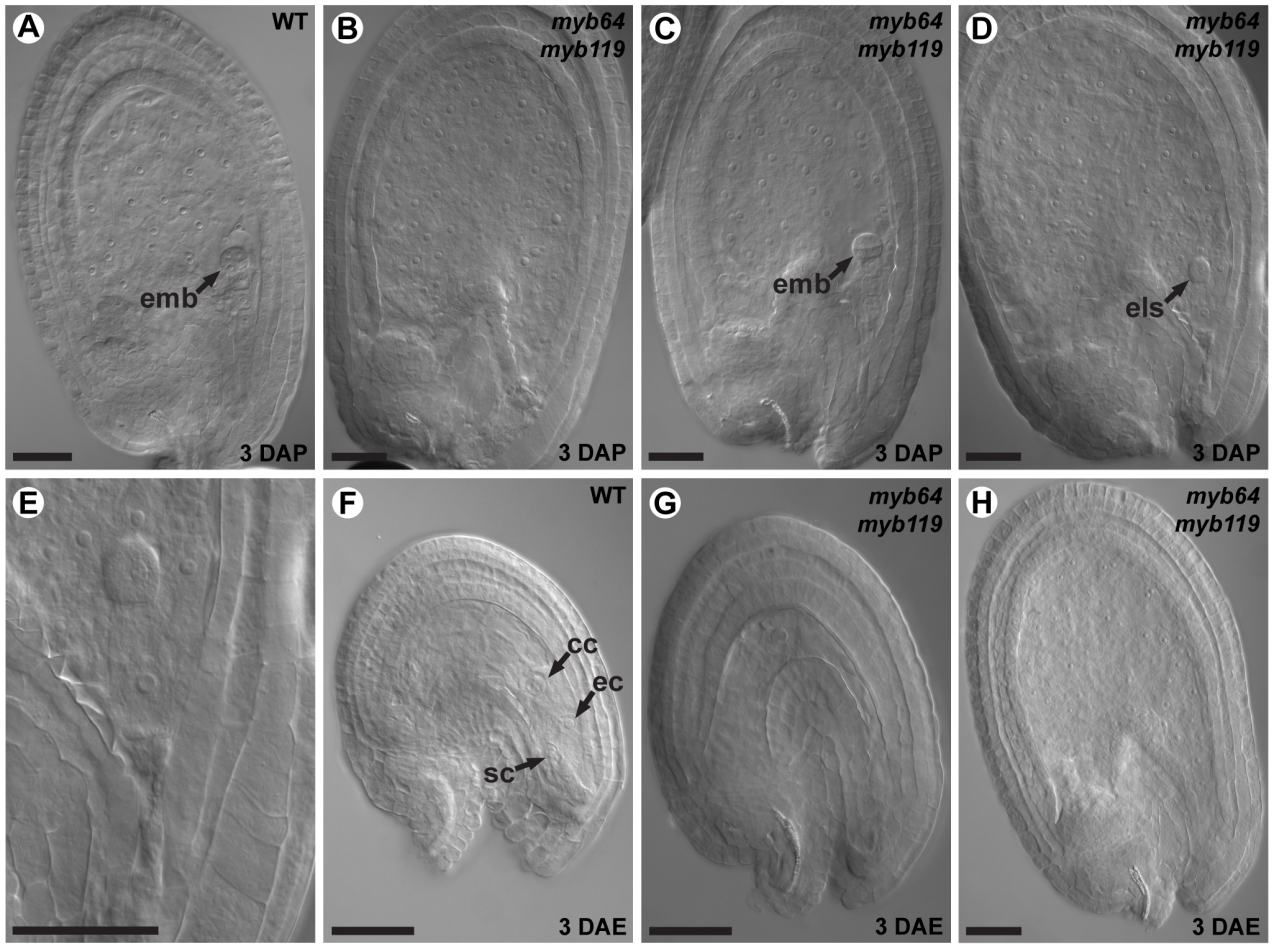
*myb64 myb119* gametophytes initiate autonomous endosperm development. (A) A wild-type seed at 3 days after pollination. Arrow points to a pre-globular stage embryo. (B) A *myb64-4 myb119-1* seed at 3 days after pollination containing proliferating endosperm but no embryo. (C) A *myb64-4 myb119-1* seed at 3 days after pollination containing proliferating endosperm and a wild-type embryo (arrow). (D) A *myb64-4 myb119-1* seed at 3 days after pollination containing proliferating endosperm and an embryo-like structure (arrow). (E) Magnification of the embryo-like structure in D. (F) A wild-type female gametophyte at 3 days after emasculation. (G) A degenerated *myb64-4 myb119-1* gametophyte at 3 days after emasculation. (H) A *myb64-4 myb119-1* autonomous seed-like structure at 3 days after emasculation. Abbreviations: ec, egg cell; emb, embryo; els, embryo-like structure; cc, central cell; DAP, days after pollination; DAE, days after emasculation; sc, synergid cell; WT, wild type. Scale bars are 40 µm.

To determine whether *myb64 myb119* double mutations affect seed development maternally, we pollinated *myb64-4 myb119-1* double-homozygous plants with wild-type pollen and analyzed cleared seeds at 3 days after pollination (DAP) (*N* = 106). Siliques resulting from this cross contained seed-like structures (Figure 5A–5E), indicating that the seed-development defect results from absence of maternal expression of *MYB64* and *MYB119*. The resulting seed-like structures fell into three categories: most (96%) lacked an embryo, but did contain tissue resembling proliferating endosperm nuclei (Figure 5B); ∼2% contained both proliferating endosperm and embryos that resembled wild-type embryos at this time point (Figure 5C); and ∼2% contained proliferating endosperm and an embryo-like structure that did not resemble any stage of wild-type embryo development and typically consisted of only a few cells (Figure 5D and 5E).

The majority of seed-like structures in *myb64 myb119* siliques were similar to autonomous seeds in mutants affected in the Fertilization Independent Seed (FIS) Polycomb Repressive Complex 2 (PRC2) [30]–[36]. To determine whether *myb64 myb119* gametophytes also initiate autonomous seed development, we emasculated flowers from *myb64-4 myb119-1* double-homozygous plants and examined the contents of the pistils at 3 days after emasculation (DAE). Wild-type pistils at 3 DAE contained only ovules with mature female gametophytes (Figure 5F). By contrast, *myb64-4 myb119-1* pistils contained a mixture of ovules with collapsed female gametophytes (81%) and seed-like structures (19%) (Figure 5G and 5H) (*N* = 437). These seed-like structures did not contain embryos or embryo-like structures, but did contain proliferating nuclei that resembled endosperm.

Additional analysis of autonomous *myb64 myb119* seed-like structures suggests that the proliferating nuclei within them have endosperm identity, as indicated by the expression of the endosperm-specific marker *ProAGL62:AGL62-GFP* (Figure S7A–S7F) [37]. They also initiate seed coat development, as indicated by vanillin staining (Figure S7G–S7I) [38], [39]. Together, these data suggest very strongly that *myb64 myb119* gametophytes produce autonomous seeds.

**Figure 6:**
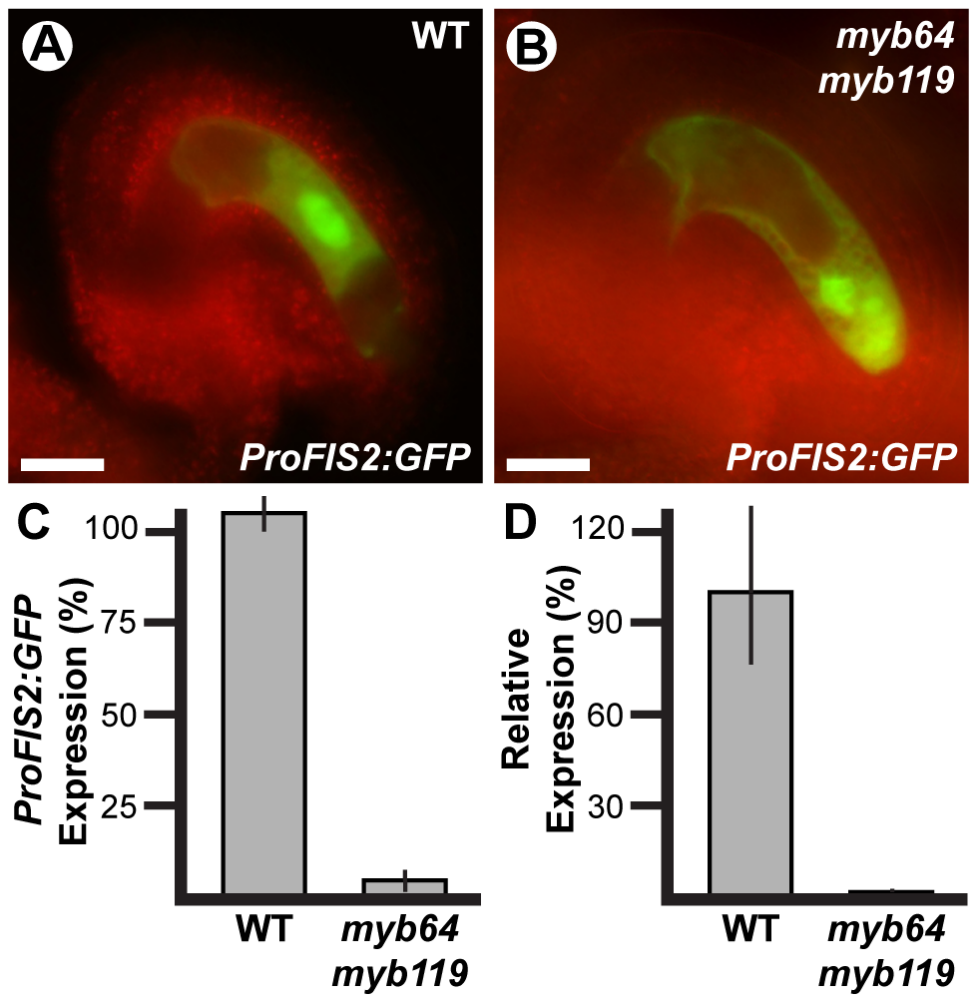
*FIS2* is downregulated in *myb64 myb119* gametophytes. (A and B) Epifluorescent micrographs of *ProFIS2:GFP* expression in wild-type (A) and *myb64-1 myb119-3* (B) gametophytes. (C) Quantification of *ProFIS2:GFP* expression in wild-type and *myb64-1 myb119-3* gametophytes. Bars represent the percentage of gametophytes expressing GFP. Wild-type expression was calculated as (GFP positive wild type)/(1/2 Total). Mutant expression was calculated as (GFP positive mutant)/(1/2 Total). (D) Relative expression of *FIS2* in wild-type and *myb64-4/myb64-4 myb119-1/myb119-1* ovaries determined by qRT-PCR. WT, wild type. Error bars indicate standard deviations. Scale bars are 25 µm.

The autonomous seed-like structures could result from absence of FIS PRC2 activity. To test this, we analyzed expression of the FIS PRC2 subunit *FIS2* in *myb64 myb119* gametophytes. We generated *myb64-1/myb64-1 myb119-3/MYB119* plants homozygous for a *FIS2* transcriptional GFP fusion (*ProFIS2:GFP*) by crossing and analyzed GFP expression at maturity (Figure 6A–6C). GFP was observed in only 5% of *myb64 myb119* gametophytes, indicating severely reduced expression (Figure 6C). When expressed in *myb64 myb119* gametophytes, *ProFIS2:GFP* expression was typically observed throughout the female gametophyte (Figure 6B), whereas in wild-type gametophytes its expression was limited to the central cell (Figure 6A). We confirmed the downregulation of *FIS2* using qRT-PCR, which showed that *FIS2* expression was strongly reduced in cDNA from *myb64-4/myb64-4 myb119-1/myb119-1* ovaries relative to wild-type ovaries (Figure 6D).

The frequency of *myb64 myb119* gametophytes expressing *ProFIS2:GFP* was reduced as compared to the other central cell marker *ProDD65:GFP* (5% versus 15%, respectively; two sample t-test: p-value = 0.03), and the difference between their frequencies roughly correlates with the number of seed-like structures observed in this genotype (Table 3), suggesting that the autonomous seeds may arise from *myb64 myb119* gametophytes with central cell identity but without a functional FIS PRC2. These data suggest that *MYB64* and *MYB119* are required to activate *FIS2* expression during the FG5 transition.

### *MYB119* expression is downregulated in *cki1* mutants

As with *myb64 myb119*, the *cki1* mutation affects stage FG5 of female gametophyte development and occasionally produces female gametophytes containing supernumerary nuclei, suggesting that *CKI1* may also be required for the FG5 transition [16]–[18]. qRT-PCR analysis of double-homozygous *myb64-4 myb119-1* ovaries indicated that *CKI1* expression was not affected in *myb64 myb119* gametophytes (Figure S8). We therefore investigated whether *MYB64* and *MYB119* are regulated through the *CKI1* pathway.

To determine if *MYB64* and *MYB119* expression is regulated by *CKI1*, we analyzed the expression of GFP fusion constructs in *cki1* mutants. Due to the transient expression of the translational fusions, we initially used transcriptional GFP reporters for both genes, which exhibited sustained expression at maturity (*ProMYB64:H2B-GFP* and *ProMYB119:H2B-GFP*). For this analysis we used *cki1-9*, which is a new *cki1* allele in the Col-0 accession that we obtained from the ABRC [27]. The T-DNA in *cki1-9* is inserted within the third exon of *CKI1* (Table S2), and CSLM analysis of *cki1-9* ovules confirmed that this allele produces an identical female gametophyte-lethal phenotype to previously reported alleles in other *Arabidopsis* accessions (Figure S8 and Table S1) [16]–[18].

**Figure 7:**
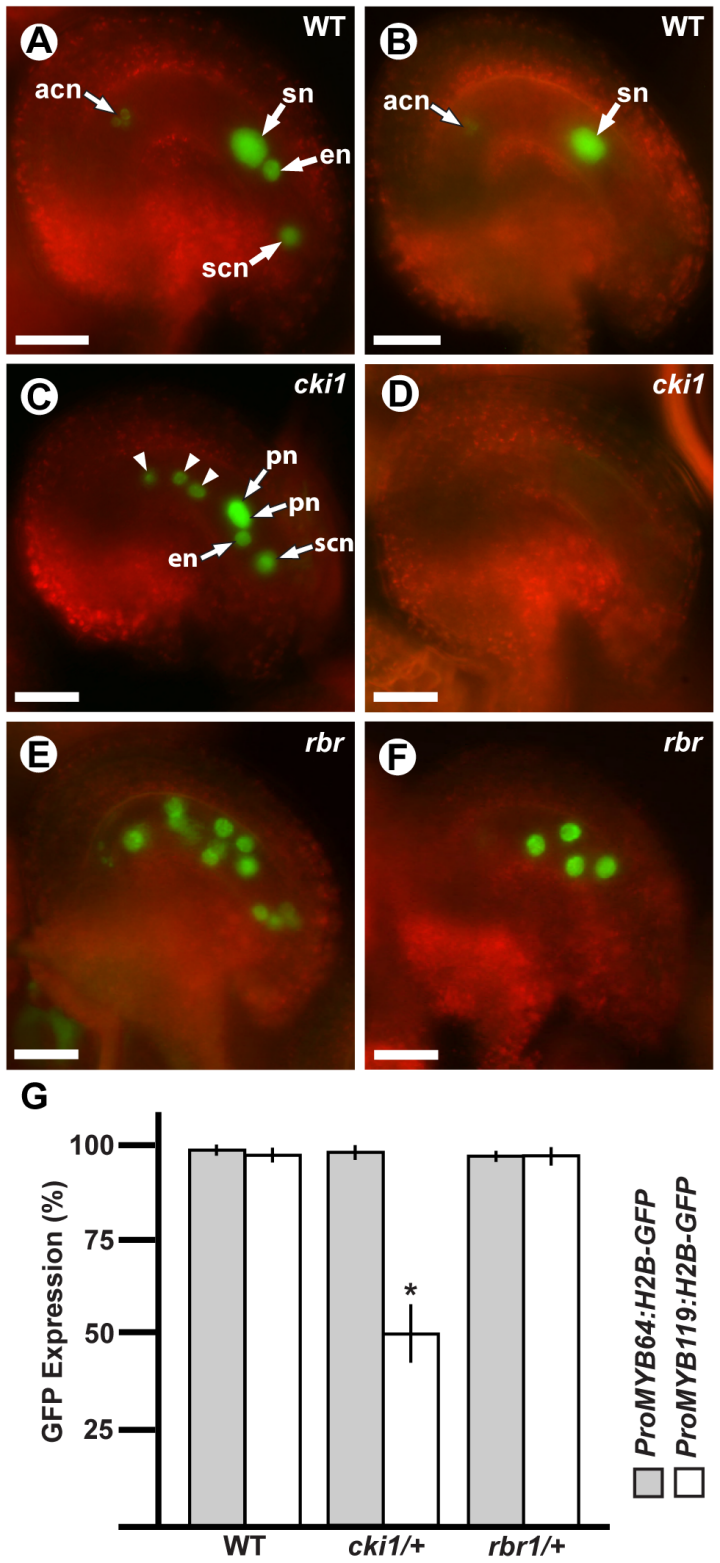
*MYB119* is downregulated in *cki1* gametophytes. (A, C and E) Epifluorescent micrographs of *ProMYB64:H2B-GFP* expression in wild-type and mutant gametophytes. (A) Expression in a wild-type female gametophyte. GFP is observed in all nuclei of the female gametophyte. (C) Expression in a *cki1-9* female gametophyte. The polar nuclei are unfused. Arrowheads indicate antipodal cell nuclei that occupy abnormal positions. (E) Expression in a multinucleate *rbr1-2* female gametophyte. (B, D and F) Epifluorescent micrographs of *ProMYB119:H2B-GFP* expression in wild-type and mutant gametophytes. (B) Expression in a wild-type female gametophyte. GFP is observed in the antipodal cell nuclei and central cell nucleus. (D) Expression in a *cki1-9* female gametophyte. *GFP* expression is not detectable in *cki1* female gametophytes. (F) Expression in a multinucleate *rbr1-2* female gametophyte. (G) Quantification of female gametophytes expressing *ProMYB64:H2B-GFP* and *ProMYB119:H2B-GFP* in wild-type (*N* = 540 and 472, respectively), *cki1-9/CKI1* (*N* = 378 and 456, respectively), and *rbr1-2/RBR* (*N* = 360 and 348 respectively) female gametophytes. Bars represent the percentage of gametophytes expressing GFP. *, p-val = 5.4E-10 for two sample t-test as compared to wild type; error bars indicate standard deviation. Abbreviations: acn, antipodal cell nuclei; en, egg cell nucleus; pn, polar nuclei of the central cell; scn, synergid cell nucleus; sn, secondary nucleus (fused polar nuclei of the central cell); WT, wild type. Scale bars are 25 µm.

Using crosses, we generated plants heterozygous for *cki1-9* and homozygous for each transcriptional GFP fusion construct, and analyzed *GFP* expression within the female gametophyte (Figure 7). In these plants, *ProMYB64:H2B-GFP* was expressed in 98% of the female gametophytes (Figure 7C and 7G). By contrast, *ProMYB119:H2B-GFP* was expressed in only 50% of the female gametophytes (Figure 7D and 7G). We obtained similar results when using the *ProMYB64:MYB64-GFP* and *ProMYB119:MYB119-GFP* translational fusions (Figure S9A–S9C), and confirmed that *MYB119* was downregulated in *cki1* mutants using qRT-PCR with cDNA from ovaries of the homozygous *cki1-8* allele (Figure S9D). These data suggest that *CKI1* is required for *MYB119* expression.

A second regulatory gene that could potentially be required for the FG5 transition is *RBR*. Similar to *myb64 myb119* mutants, *rbr* mutant female gametophytes contain supernumerary nuclei or cells and also exhibit defects in differentiation [5], [6], [9]. *RBR* is expressed before *MYB64* and *MYB119* during early female gametogenesis [6], suggesting that *RBR* may be required for *MYB64* and *MYB119* expression. To test this, we crossed *ProMYB64:H2B-GFP* and *ProMYB119:H2B-GFP* into *rbr1-2* plants, and analyzed plants heterozygous for *rbr1-2* and homozygous for each transcriptional GFP fusion. GFP expression for both constructs was unaffected in the *rbr1-2* mutant (Figure 7E–7G), suggesting that RBR does not regulate *MYB64* or *MYB119* expression in the female gametophyte.

### *cki1 myb64* gametophytes fail to exit coenocytic development

The above results suggest that *MYB119* is regulated through the CKI1 pathway. If this is true, *cki1 myb64* double-mutants should exhibit a phenotype similar to that of *myb64 myb119* double-mutants. To test this, we generated double- and triple-mutant female gametophytes and analyzed their phenotypes using CSLM.

**Figure 8:**
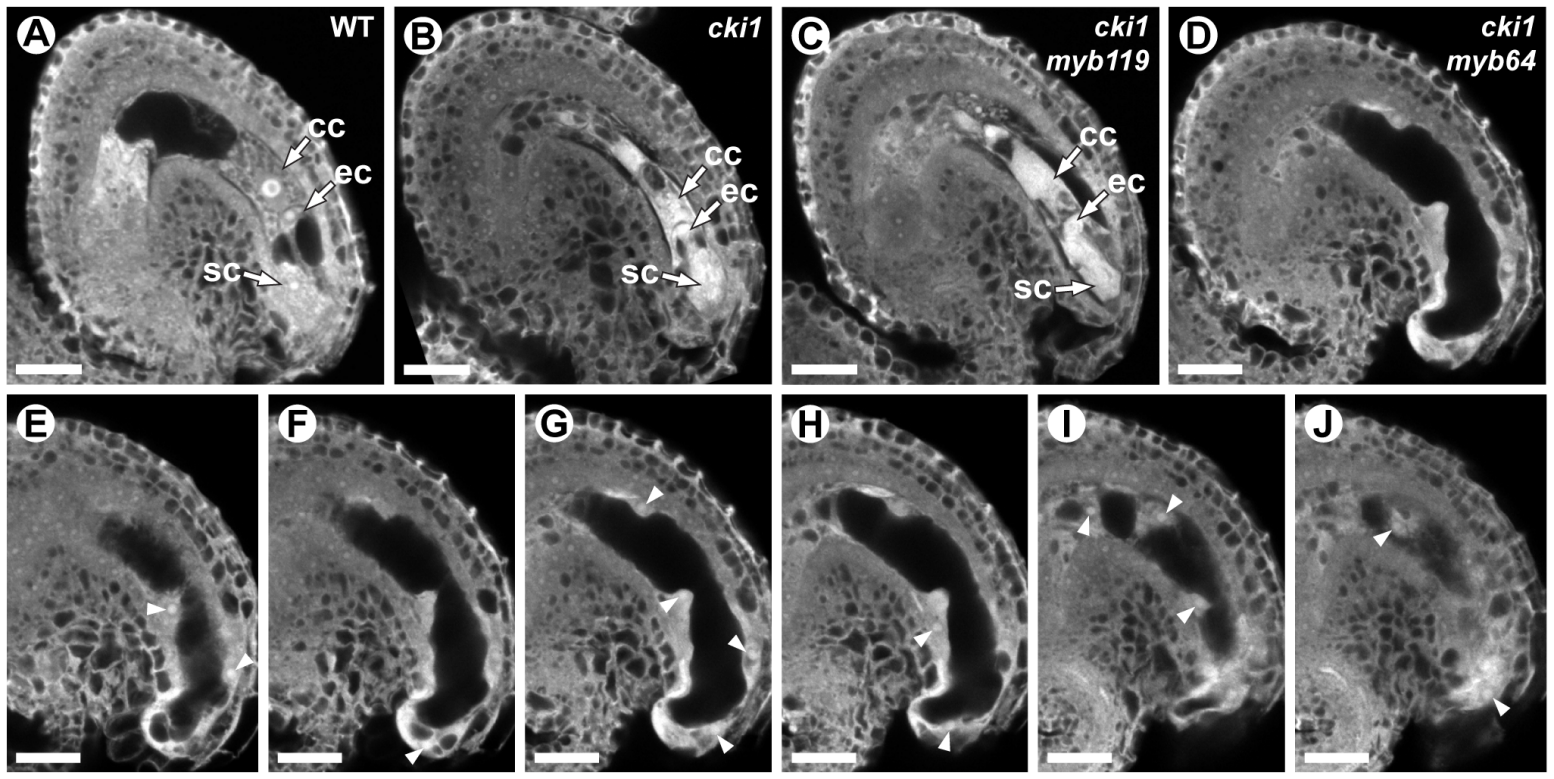
*cki1 myb64* double mutant gametophytes fail to initiate the FG5 transition. (A) A mature wild-type female gametophyte at stage FG7. (B) A *cki1-9* female gametophyte at maturity. The central cell and antipodal cells are degenerated, whereas positioning and cellularization of the egg and synergid cells are unaffected. (C) A *cki1-9 myb119-3* double mutant female gametophyte. *cki1 myb119* gametophytes resemble *cki1* single mutant gametophytes. (D) A *cki1-9 myb64-1* female gametophyte. *cki1 myb64* gametophytes are enlarged, contain supernumerary nuclei and resemble *myb64 myb119* gametophytes. (E–J) A Z-stack series of the ovule depicted in D. Arrowheads indicate fourteen nuclei. Abbreviations: cc, central cell; ec, egg cell; sc, synergid cell; WT, wild type. Scale bars are 20 µm.

We analyzed double-mutant female gametophytes in *cki1-9/CKI1 myb119-3/myb119-3* and *cki1-9/CKI1 myb64-1/myb64-1* plants. In both genotypes, ∼50% of the female gametophytes were defective (Figure 8). *cki1 myb119* gametophytes were indistinguishable from *cki1* gametophytes (Figure 8B and 8C) (*N* = 229). By contrast, most (69%) *cki1 myb64* gametophytes did not resemble *cki1* gametophytes but instead resembled *myb64 myb119* gametophytes: 47% of *cki1 myb64* gametophytes were enlarged, protruded from the micropyle of the ovule, and contained supernumerary nuclei (Figure 8D–8J); and 22% were collapsed or cellularized in a manner similar to *myb64 myb119* gametophytes. The remaining 31% of *cki1 myb64* gametophytes resembled *cki1* gametophytes (*N* = 311). These results are consistent with our expression data showing that *MYB119* is downregulated in *cki1* female gametophytes.

We also analyzed triple-mutant female gametophytes in *cki1-9/CKI1 myb64-1/MYB64 myb119-3/myb119-3* plants. Pistils from these plants contain female gametophytes with four different genotypes: 25% *CKI1 MYB64 myb119*, 25% *CKI1 myb64 myb119*, 25% *cki1 MYB64 myb119*, and 25% *cki1 myb64 myb119*. As expected, pistils from triple mutant plants contained ∼75% defective gametophytes. Of the total gametophytes examined, 24% resembled *cki1* gametophytes while 53% resembled gametophytes from *myb64 myb119* plants (*N* = 139). Therefore, *cki1 myb64 myb119* gametophytes resemble *myb64 myb119* gametophytes, whereas *cki1 MYB64 myb119* gametophytes resemble *cki1* gametophytes. These results demonstrate that *MYB64* has activity in *cki1 myb119* gametophytes, which is consistent with our expression data showing that *MYB64* is expressed in *cki1* gametophytes.

### *CKI1* is expressed throughout female gametophyte development

If *MYB119* expression is regulated through the CKI1 pathway, these two genes should be co-expressed. To test this, we analyzed transgenic lines containing a translational fusion construct (*ProCKI1:CKI1-GFP*). *ProCKI1:CKI1-GFP* was capable of complementing the *cki1-9/CKI1* and *cki1-9/CKI1 myb64-1/myb64-1* silique phenotypes (Table S1). This analysis also allowed us to determine the subcellular localization of CKI1 within the female gametophyte; although CKI1 has been shown to localize to the plasma membrane when ectopically expressed in *Arabidopsis* protoplasts [10], localization within the developing gametophyte has not been determined.

**Figure 9:**
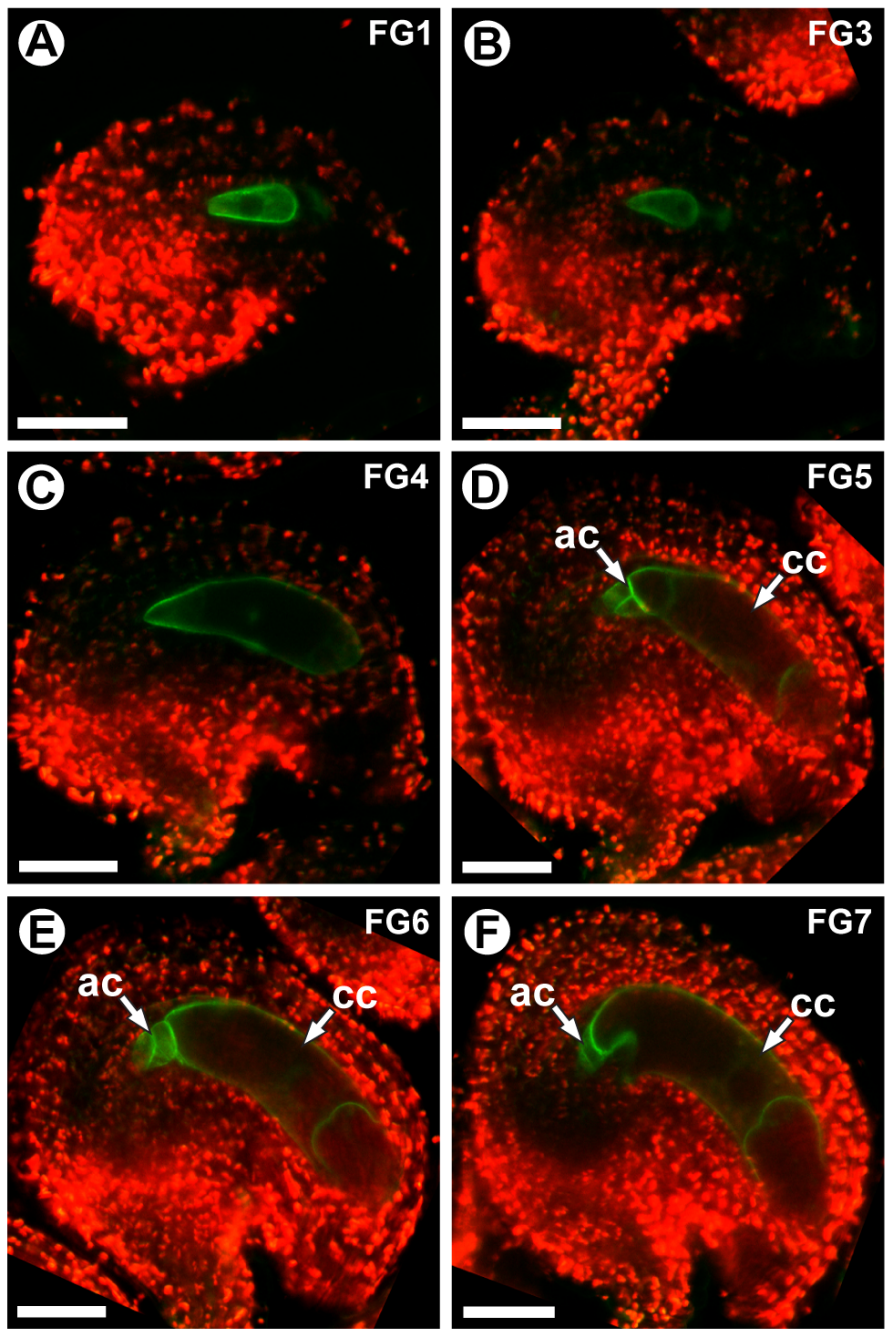
*CKI1* expression is restricted to the chalazal region during the FG5 transition. CSLM micrographs of *ProCKI1:CKI1-GFP* expression in wild-type gametophytes. CKI1-GFP is localized primarily to the plasma membrane within the female gametophyte (A–F). Weak cytoplasmic localization is also visible during stages FG1 (A) and FG3 (B). During coenocytic development (stages FG1–FG4), *ProCKI1:CKI1-GFP* is expressed throughout the female gametophyte (A–C). Post-cellularization (stages FG5–FG7), CKI1-GFP expression becomes restricted to the central cell and antipodal cells (D–F). Abbreviations: ac, antipodal cells; cc, central cell. Scale bars are 25 µm.

*ProCKI1:CKI1-GFP* expression was detectable during all stages of female gametophyte development (stages FG1–FG7). Before cellularization (stages FG1–FG4), expression was detected throughout the gametophyte (Figure 9A–9C). During these stages, CKI1-GFP was primarily localized to the plasma membrane; however during stages FG1–FG3 weak cytoplasmic localization was also detected (Figure 9A and 9B). Post-cellularization (stages FG5–FG7), *ProCKI1:CKI1-GFP* expression was restricted to the three antipodal cells and the central cell, and CKI1-GFP was primarily localized to the plasma membrane (Figure 9D–9F). The post-cellularization expression of *CKI1* is consistent with the reported phenotype of *cki1* gametophytes, which primarily exhibit defects in the chalazal region of the female gametophyte including improper positioning of the antipodal cell nuclei, unfused polar nuclei, and degeneration of the central cell [17] (Figure 7C and Figure 8B). These data show that *MYB119* and *CKI1* are co-expressed during stages FG4–FG6 of female gametophyte development. Additionally, *CKI1* expression within the gametophyte becomes polarized during the FG5 transition, indicating that CKI1-dependent TCS activity is restricted to the chalazal pole.

## Discussion

During wild-type female gametogenesis, the embryo sac initially develops coenocytically, during stages FG1–FG4. Then, during the FG5 transition, the coenocytic pattern ceases and the developing embryo sac cellularizes. Concomitantly, nuclear division ceases, cell expansion attenuates, and the resulting cells differentiate. *myb64 myb119* female gametophytes are defective in all aspects of the FG5 transition. Most *myb64 myb119* gametophytes continue the coenocytic developmental pattern at stage FG5 and fail to cellularize, cease nuclear division, and attenuate cell growth, resulting in enlarged coenocytes with supernumerary nuclei (Figure 3). Furthermore, in cases where *myb64 myb119* gametophytes do cellularize, they contain extra cells and the resulting cells are defective in cellular differentiation, as indicated by reduced expression of cell-type specific markers (Figure 4). As putative transcription factors, it is likely that MYB64 and MYB119 function to regulate a large number of genes required for the multiple processes that occur during the FG5 transition of female gametogenesis, including cell growth, cellularization, differentiation, and cell cycle regulation. The regulation and timing of *MYB64* and *MYB119* expression is therefore a critical step in formation of female gametes.

We have shown that *MYB119* expression is downregulated in *cki1* female gametophytes. This conclusion is supported by both expression (Figure 7 and Figure S9) and genetic (Figure 8) data. CKI1 is the primary activator of TCS within the female gametophyte, as none of the known cytokinin receptors (AHK2–AHK4) are necessary for female gametogenesis [18]–[23]. Although CKI1 is required for female gametophyte development [16]–[18], the specific developmental processes it regulates are largely unknown. We have shown that at least one of these processes is to promote the FG5 transition through the regulation of *MYB119*; however, whether *MYB119* is a direct target of the CKI1-TCS pathway has yet to be determined.

**Figure 10:**
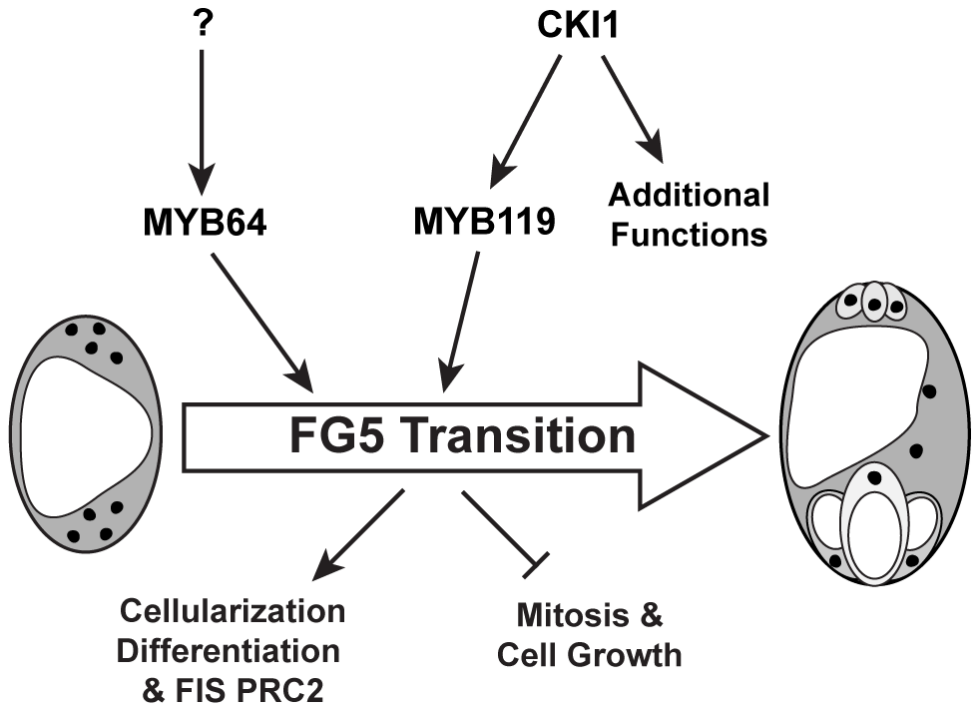
Regulation of the FG5 transition during female gametogenesis. MYB64 and MYB119 redundantly regulate the FG5 transition. *MYB119* expression is regulated by CKI1 whereas the regulation of *MYB64* has yet to be determined. During the FG5 transition the embryo sac cellularizes, nuclear division ceases, cell growth attenuates, and the resulting cells differentiate.

Although *MYB64* acts redundantly with *MYB119*, expression of *MYB64* is not affected in *cki1* gametophytes, suggesting that it is independently regulated. This conclusion is supported by both expression (Figure 7 and Figure S9) and genetic (Figure 8) data. Independent regulation of *MYB64* and *MYB119* can also be observed in their slightly different expression patterns (Figure 1). These data suggest that two parallel, yet redundant pathways exist to promote the FG5 transition in *Arabidopsis*. One pathway involves *MYB119*, which is regulated by CKI1, and a second pathway involves *MYB64*, which is regulated by an as yet unknown regulator (Figure 10).

Although *cki1* mutants arrest development during stage FG5, our data indicate that the *cki1* single mutant phenotype results from functions of CKI1 that are independent of *MYB119* downregulation. First, MYB119 and MYB64 are functionally redundant proteins but only *MYB119* is downregulated in *cki1* mutants (Figure 7 and Figure S9). Second, *myb119* gametophytes are phenotypically wild type, indicating the *cki1* phenotype is not due to downregulation of *MYB119* (Figure S3). Third, our triple mutant analysis demonstrates that *MYB64* has activity in *cki1 myb119* gametophytes. Together these data suggest that *MYB64* expression is sufficient to initiate the FG5 transition in the absence of CKI1 or MYB119. Consistent with our data, *cki1* mutants typically contain synergid and egg cell structures (Figure 8B) [17].

The female gametophyte is a highly polarized structure consisting of the egg and synergid cells at the micropylar pole, and the antipodal cells at the chalazal pole. Gametophytic polarity within *myb64 myb119* gametophytes is defective; specifically, *myb64 myb119* gametophytes exhibit an expansion of chalazal cell identity and a loss of micropylar cell identity (Figure 4). Establishment of polarity within the female gametophyte is poorly understood. However, several lines of evidence suggest that nuclear positioning within the coenocytic gametophyte is a primary determinate of cell fate [5], [6], [40]–[43] and that positional information is conveyed through an asymmetric gradient of the plant hormone auxin emanating from the micropylar pole [44]. Initiation of the micropylar auxin gradient is reported to occur very early (stages FG1–FG3) whereas *MYB64* and *MYB119* expression is not observed until stage FG4; therefore, it is unlikely that these genes are required for establishment of the auxin gradient. However, MYB64 and MYB119 could be required to interpret this positional information prior to cellularization. Alternatively, the micropylar auxin gradient may be disrupted in *myb64 myb119* gametophytes due to their prolonged coenocytic development.

CKI1 activates the cytokinin TCS pathway independent of cytokinin [10], [12]–[15], [45]; therefore, *CKI1* expression likely represents areas of TCS activity. During the FG5 transition, *CKI1* expression becomes restricted to the chalazal-most cells of the female gametophyte (antipodal cells and central cell) (Figure 9), suggesting the existence of polarized CKI1-dependent TCS activity within the female gametophyte. Consistent with the observed *CKI1-GFP* expression pattern, mutations in *CKI1* primarily affect the central cell and antipodal cells [17] (Figure 7B and Figure 8B). Notably *CKI1* is expressed at the opposite pole from which an auxin source within the female gametophyte is initiated [44]. An antagonizing role between auxin and cytokinin-dependent TCS has been documented during a number of key developmental steps in *Arabidopsis* [46], suggesting that interactions between chalazal CKI1-dependent TCS and a micropylar auxin source may play a role in regulating the FG5 transition.

In most plant species, seed development initiates only following fertilization. In the absence of fertilization, the FIS PRC2 represses initiation of endosperm development within the central cell, and gametophytes without a functional FIS PRC2 initiate endosperm development in the absence of fertilization [30]–[36]. *myb64 myb119* gametophytes also give rise to seed-like structures in the absence of fertilization (Figure 5, Figure S6 and Figure S7), suggesting that *MYB64* and *MYB119* are required to activate FIS PRC2 activity within the central cell during the FG5 transition. Consistent with this, expression of the FIS PRC2 subunit FIS2 is reduced in *myb64 myb119* gametophytes (Figure 6), indicating that a functional FIS PRC2 is not present. *MYB64* and *MYB119* are expressed transiently during female gametogenesis (Figure 1); therefore, it is unlikely that they directly regulate *FIS2* expression. For example, regulation of *FIS2* by MYB64 and MYB119 may act through *DEMETER* and/or *DNA METHYLTRANSFERASE 1*, which are required for *FIS2* activation or repression, respectively [7], [47]–[49]. Further experiments will be required to place *MYB64* and *MYB119* within this pathway.

In summary, the results presented here indicate that MYB64 and MYB119 act redundantly as regulators of the FG5 transition and are independently regulated. *MYB119* is regulated by CKI1 whereas the regulator of *MYB64* has yet to be determined. During the FG5 transition, MYB64 and MYB119 regulate multiple developmental processes including cell growth, nuclear divisions, cellularization, differentiation and activation of the PRC2 subunit *FIS2* (Figure 10).

## Materials and Methods

### Plant material and growth conditions

All *Arabidopsis thaliana (L.) Heynh* plants used were derived from the Columbia (Col-0 or Col-3) and Wassilewskija (Ws) accessions. Seeds from *myb64-1* (Col-0), *myb64-2* (Col-0), *myb64-3* (Col-3), *myb64-4* (Col-3), *myb119-1* (Col-0), *myb119-2* (Col-0), *myb119-3* (Col-0), *myb119-4* (Col-3), *myb119-5* (Col-3), *cki1-9* (Col-0) and Ws (Stock # CS28823) plants were obtained from the *Arabidopsis* Biological Resource Center. Seeds from *rbr1-2* (Col-0) plants were kindly provided by Frédéric Berger. Seeds from *ProFIS2:GFP* (Col-0) plants were kindly provided by Ramin Yadegari. Seeds from *cki1-8* (Ws) plants were kindly provided by Jianru Zou. T-DNA borders for *myb64-1*, *myb64-4*, *myb119-1*, *myb119-3*, *myb119-5* and *cki1-9* were determined by amplifying the borders with either standard PCR or inverse PCR followed by sequencing. T-DNA borders and primer sequences used for amplification are listed in Table S2 and Table S3, respectively. Genotypes were determined by standard PCR reactions using primers listed in Table S3. Seeds were surface sterilized with chloride gas and sown on 0.5X MS salts, 0.05% MES, 1% sucrose and 0.8% Phytagar. For T1 selection, the appropriate selective agent was also added to the media. Seedlings were transferred to soil after 12 days of growth. All plants were grown at 20°C under 24-hour illumination.

### Constructs and transformation

*ProMYB64:MYB64-GFP*, *ProMYB119:MYB119-GFP* and *ProCKI1:CKI1-GFP* were generated by amplifying ∼2 kb of upstream sequence and the full gene coding sequence minus the stop codon from Col-0 genomic DNA using the primers listed in Table S3. The PCR fragments were then cloned into the pENTR/D-TOPO vector (Invitrogen). pENTR/D-TOPO clones were then recombined into the destination vector pGWB450 [50] using LR Clonase II (Invitrogen). Approximately 1.0 kb of 3′ sequence from *MYB119* was amplified using the primers listed in Table S3 and cloned into *ProMYB119:MYB119-GFP* at a SacI site 3′ of the *GFP* coding region. *ProMYB64:H2B-GFP* and *ProMYB119:H2B-GFP* were generated by amplifying ∼2.0 kb of upstream sequence from Col-0 genomic DNA using primers listed in Table S3. The PCR products were digested with appropriate restriction enzymes and ligated into the pBI-n1gfp vector [24]. Plants were transformed with *Agrobacterium tumefaciens* (GV3101-pMP90) using a modified floral dip procedure [51]. For all constructs multiple independent T1 plants were analyzed, and are summarized in Table S4.

### qRT-PCR analysis

Pistils were emasculated at stage 12c prior to collection. For qRT-PCR analysis of *MYB64* and *MYB119* in wild type, pistils were collected 24 hours after emasculation. For qRT-PCR analysis of *CKI1*, *FIS2*, and *MYB119* in *myb64-4 myb119-1* double-homozygotes or *cki1-8* homozygotes, pistils were collected 12–16 hours after emasculation. Siliques were collected 36 hours after pollination of emasculated flowers. Whole seedlings were germinated on GM and collected 10 days after germination. Stamens were collected from stage 14 flowers. All tissue was immediately frozen in liquid nitrogen. RNA was extracted using the RNeasy Mini Kit (Qiagen). cDNA was transcribed from 1 µg of total RNA using the QuantiTect Reverse Transcription Kit (Qiagen). qRT-PCR was performed using SYBR Green with the primers listed in Table S3. Relative expression was calculated according to the ΔΔC_T_ method, with the average of three biological replicates normalized to *ACTIN2* reported, unless otherwise noted in the figure legend.

### Microscopy

For epifluorescence microscopy, tissue was dissected in water and analyzed using a Zeiss Axioplan compound microscope with DIC and epifluorescent optics. Mature pollen was stained with 4′,6-diamidino-2-phenylindole (DAPI) as previously described [52]. For confocal fluorescence microscopy, tissue was dissected in water and analyzed using a Zeiss LSM 510 microscope. Analysis of *ProMYB64:H2B-GFP* and *ProMYB119:H2B-GFP* in *cki1* and *rbr* mutants was done by emasculating stage 12c flowers and examining the ovules 16 hours later. Analysis of *ProMYB64:MYB64-GFP* and *ProMYB119:MYB119-GFP* in *cki1* mutants was done by emasculating stage 12c flowers and examining the ovules 12 hours later.

For CSLM analysis of gametophyte development, pistils were sliced open along the replum using a needle and immersed in fixative containing 1×PBS and 4% glutaraldehyde. Tissue was fixed at room temperature for 2.5 hours under vacuum, followed by an ethanol dehydration series for 15 minutes each: 1×PBS 10% ethanol, 1×PBS 20% ethanol, 1×PBS 40% ethanol, 0.5×PBS 60% ethanol, 80% ethanol. Tissue was incubated in 95% ethanol overnight followed by two 30 min. incubations in 100% ethanol. Tissue was cleared in 2∶1 Benzyl Benzoate∶Benzyl Alcohol for 30 minutes. After rinsing pistils off in immersion oil, ovules were dissected directly into a drop of immersion oil and the coverslip was secured with nail polish. Ovules were then imaged using a Zeiss LSM 510 as previously described [53].

Seeds were cleared by incubating siliques, opened along the replum, in 9∶1 Ethanol∶Acetic acid for 2 hours, followed by two washes in 90% ethanol for 30 minutes each. Seeds were dissected out directly into a drop of chloral hydrate (chloral hydrate∶water∶glycerol (8∶2∶1)). Cleared tissue was imaged using a Zeiss Axioplan compound microscope with DIC optics.

For vanillin staining, pistils or siliques were sliced open along the replum using a needle and immersed in 1% (w/v) vanillin, 6N HCl for 30 minutes under vacuum. Carpels were then removed and ovules or seeds were imaged using an Olympus BX50 compound microscope with DIC optics.

## Supporting Information

Figure S1 Sporophytic expression of *ProMYB64:H2B-GFP* and *ProMYB119:H2B-GFP*. (A and B) *ProMYB64:H2B-GFP* (A) and *ProMYB119:H2B-GFP* (B) expression in the septum of the ovary. (C) *ProMYB119:H2B-GFP* expression in the filament of a stamen. Strong autofluorescent signal in the anther does not represent GFP signal. Sale bars are 50 µm.
(TIF)

Figure S2 *MYB64* and *MYB119* gene structures. Both *MYB64* and *MYB119* consist of three exons (gray boxes) and two introns. The coding domain sequences of *MYB64* and *MYB119* are predicted to encode R2R3-MYB related proteins that share ∼65% amino acid identity. The insertion sites of the T-DNAs used are indicated by triangles.
(TIF)

Figure S3 Phenotype of *myb64-1* and *myb119-3* gametophytes. (A–C) CSLM micrographs of wild-type (A), *myb64-1* (B) and *myb119-3* (C) mature female gametophytes. *myb64-1* and *myb119-3* female gametophytes exhibit wild-type morphology. cc, central cell; ec, egg cell; sc, synergid cell; WT, wild type. Scale bars are 20 µm.
(TIF)

Figure S4 Analysis of *myb64 myb119* pollen. Examples of pollen grains from wild-type (A and B), and *myb64-1/MYB64 myb119-3/myb119-3* (C and D) plants stained with DAPI. Sperm cell nuclei are indicated by arrowheads. Vegetative nuclei are indicated by arrows. WT, wild type. Scale bars are 10 µm.
(TIF)

Figure S5 Additional Z-stack series of *myb64 myb119* gametophytes. (A–E) A Z-stack series of the coenocytic, eight-nucleate *myb64 myb119* female gametophyte depicted in Figure 3E. Eight nuclei are indicated by arrowheads. (F–J) A Z-stack series of the cellularized *myb64 myb119* female gametophyte depicted in Figure 3G. Thirteen nuclei are indicated by arrowheads. Scale bars are 20 µm.
(TIF)

Figure S6 Silique phenotype of *myb64 myb119* plants. (A and B) Opened siliques from a self-fertilized wild-type plant (A) and a self-fertilized *myb64-1/MYB64 myb119-3/myb119-3* plant (B). Arrowheads indicate desiccated ovules and asterisks indicate white or collapsed seed-like structures.
(TIF)

Figure S7 Autonomous endosperm and seed coat development in *myb64 myb119* gametophytes. (A–F) Analysis of *ProAGL62:AGL62-GFP* expression in wild-type and *myb64 myb119* plants. (A and D) Expression in wild-type seeds at 3 days after emasculation. AGL62-GFP is not detected (*N* = 257). (B and E) Expression in *myb64 myb119* autonomous seed at 3 days after emasculation. AGL62-GFP is detected in the proliferating nuclei. AGL62-GFP expression was observed in 6% (+/−1.7%) of *myb64-1/MYB64 myb119-3/myb119-3* ovules at 3 days after emasculation (*N* = 353). (C and F) Expression in wild-type seeds at 3 days after pollination. AGL62-GFP is detected in proliferating endosperm. (G–I) Vanillin staining of proanthocyanidin accumulation. Proanthocyanidins accumulate in the endothelium layer of the seed coat post-fertilization, and stain dark red in the presence of vanillin in low pH [38], [39]. (G) Wild-type ovules at 3 days after emasculation. Ovules at this stage do not stain positive for proanthocyanidins (*N* = 351). (H) *myb64-4/myb64-4 myb119-1/myb119-1* ovules at 3 days after emasculation. Staining was observed in 49% (+/−9.4%) of *myb64-4/myb64-4 myb119-1/myb119-1* ovules (*N* = 699). (I) A wild-type seed at 3 days after pollination. DAE, days after emasculation; DAP, days after pollination; WT, wild-type. Scale bars are 40 µm.
(TIF)

Figure S8 Analysis of the *cki1-9* allele and *CKI1* expression in *myb64 myb119* gametophytes. (A–C) CSLM micrographs of wild-type (A), and *cki1-9* (B and C) female gametophytes. (B) An example of the typical *cki1* female gametophyte phenotype. The central cell and antipodal cells are degenerated, whereas positioning and cellularization of the egg and synergid cells are unaffected. (C) An example of the less frequent multinucleate/multivacuolate *cki1* female gametophyte phenotype. (D) Relative expression of *CKI1* in wild-type and *myb64-4/myb64-4 myb119-1/myb119-1* ovaries as determined by qRT-PCR. Abbreviations: cc, central cell; ec, egg cell; sc, synergid cell. Error bars indicate standard deviations. WT, wild type. Scale bars are 20 µm.
(TIF)

Figure S9 Analysis of *MYB64* and *MYB119* expression in *cki1* female gametophytes. (A and B) Epifluorescent micrographs of *ProMYB64:MYB64-GFP* expression in wild-type (A) and *cki1-9* (B) female gametophytes. Indeterminate nuclei are indicated by arrowheads. (C) Percentage of female gametophytes expressing *ProMYB64:MYB64-GFP* and *ProMYB119:MYB119-GFP* in wild type (*N* = 483 and 608, respectively) and *cki1-9/CKI1* (*N* = 301 and 407, respectively). *, p-val = 3.1E-12 for a two sample t-test as compared to wild type. (D) Relative expression of *MYB119* in wild-type and *cki1-8/cki1-8* ovaries as determined by qRT-PCR. The average of three biological and three technical replicates normalized to *ACTIN2* is reported. We observed a moderate, but significant reduction of *MYB119* expression in *cki1-8* ovaries. Our ability to detect *MYB119* downregulation in this experiment was limited by sporophytic expression of *MYB119* in ovaries (Figure S1B), and may reflect differences in accessions (*cki1-9* is in the Col-0 background, while *cki1-8* is in the Ws background). **, p-val = 6.7E-4 for a two sample t-test as compared to wild type. sn, secondary nucleus of the central cell; en, egg cell nucleus; WT, wild type.
(TIF)

Table S1 Molecular complementation of *myb64 myb119* and *cki1* seed phenotypes.
(XLSX)

Table S2 Summary of *myb64*, *myb119* and *cki1* alleles.
(XLSX)

Table S3 List of primers used in this study.
(XLSX)

Table S4 Summary of transformants analyzed for transcriptional and translational GFP fusions.
(XLSX)
