# Supplementary figures and images for: MYB64 and MYB119 Are Required for Cellularization and Differentiation during Female Gametogenesis in Arabidopsis thaliana
Source: PLoS Genet. 2013 Sep 19;9(9):e1003783. doi: 10.1371/journal.pgen.1003783 (PMC3778002; doi:10.1371/journal.pgen.1003783)

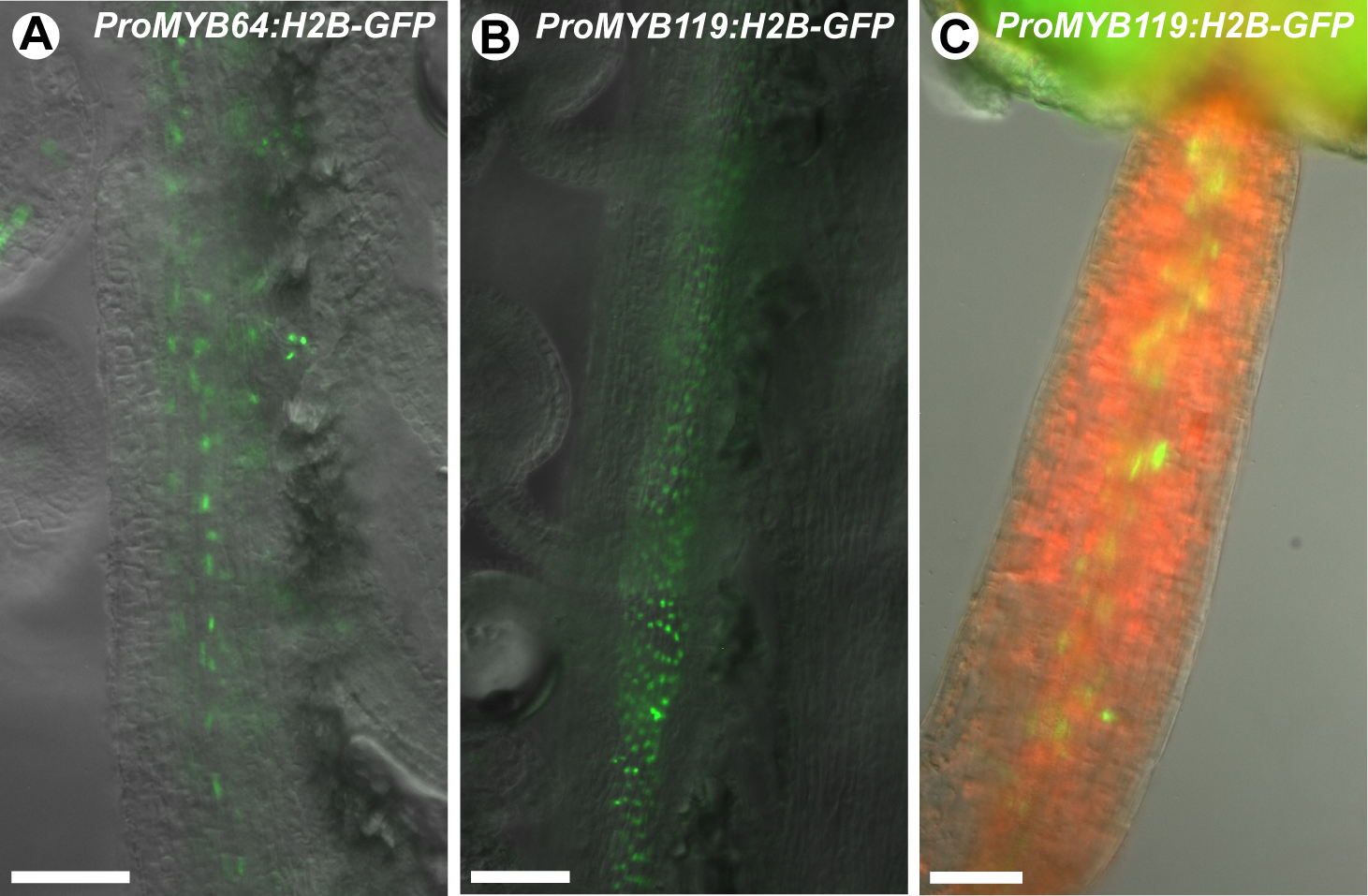

Supplement: Figure S1 — Sporophytic expression of ProMYB64:H2B-GFP and ProMYB119:H2B-GFP. (A and B) ProMYB64:H2B-GFP (A) and ProMYB119:H2B-GFP (B) expression in the septum of the ovary. (C) ProMYB119:H2B-GFP expression in the filament of a stamen. Strong autofluorescent signal in the anther does not represent GFP signal. Sale bars are 50 µm. (TIF) [file pgen.1003783.s001.tif]

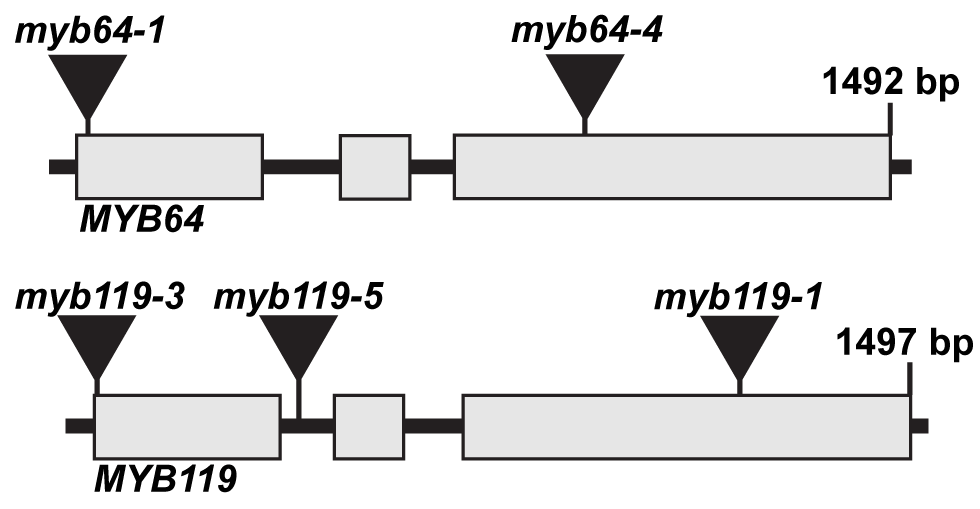

Supplement: Figure S2 — MYB64 and MYB119 gene structures. Both MYB64 and MYB119 consist of three exons (gray boxes) and two introns. The coding domain sequences of MYB64 and MYB119 are predicted to encode R2R3-MYB related proteins that share ∼65% amino acid identity. The insertion sites of the T-DNAs used are indicated by triangles. (TIF) [file pgen.1003783.s002.tif]

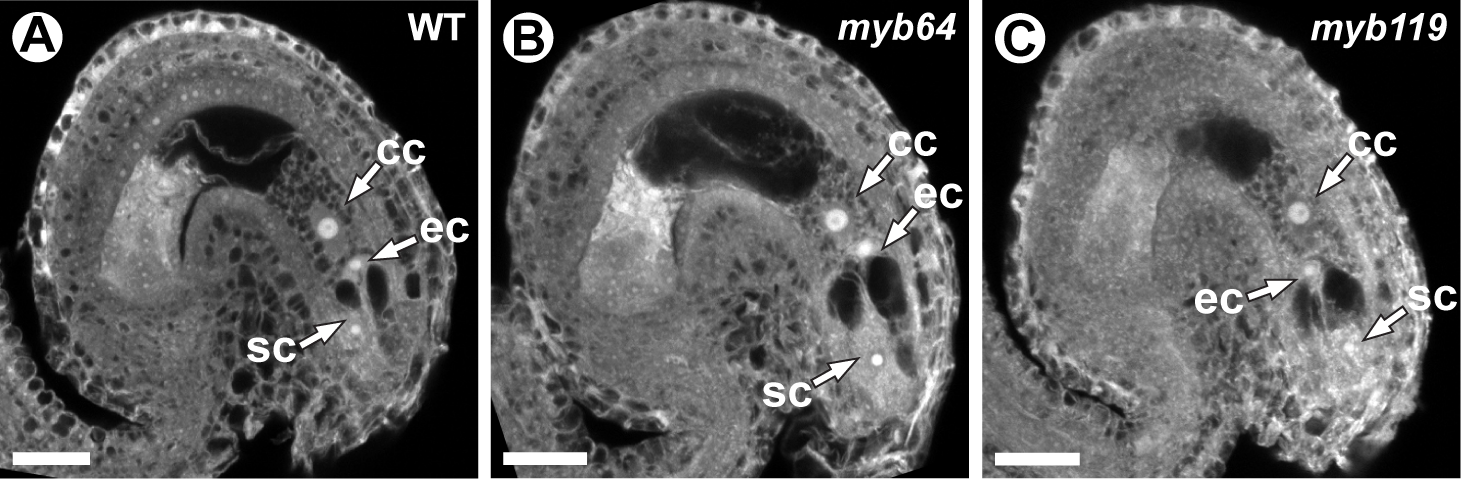

Supplement: Figure S3 — Phenotype of myb64-1 and myb119-3 gametophytes. (A–C) CSLM micrographs of wild-type (A), myb64-1 (B) and myb119-3 (C) mature female gametophytes. myb64-1 and myb119-3 female gametophytes exhibit wild-type morphology. cc, central cell; ec, egg cell; sc, synergid cell; WT, wild type. Scale bars are 20 µm. (TIF) [file pgen.1003783.s003.tif]

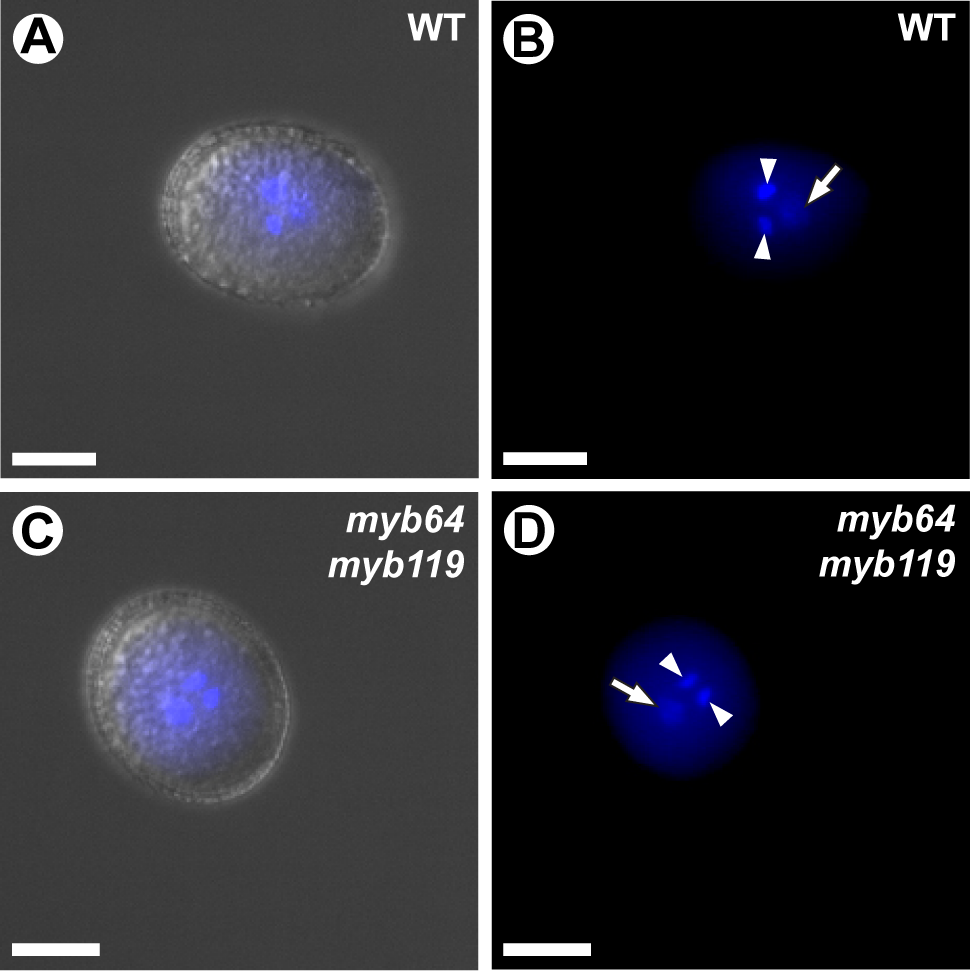

Supplement: Figure S4 — Analysis of myb64 myb119 pollen. Examples of pollen grains from wild-type (A and B), and myb64-1/MYB64 myb119-3/myb119-3 (C and D) plants stained with DAPI. Sperm cell nuclei are indicated by arrowheads. Vegetative nuclei are indicated by arrows. WT, wild type. Scale bars are 10 µm. (TIF) [file pgen.1003783.s004.tif]

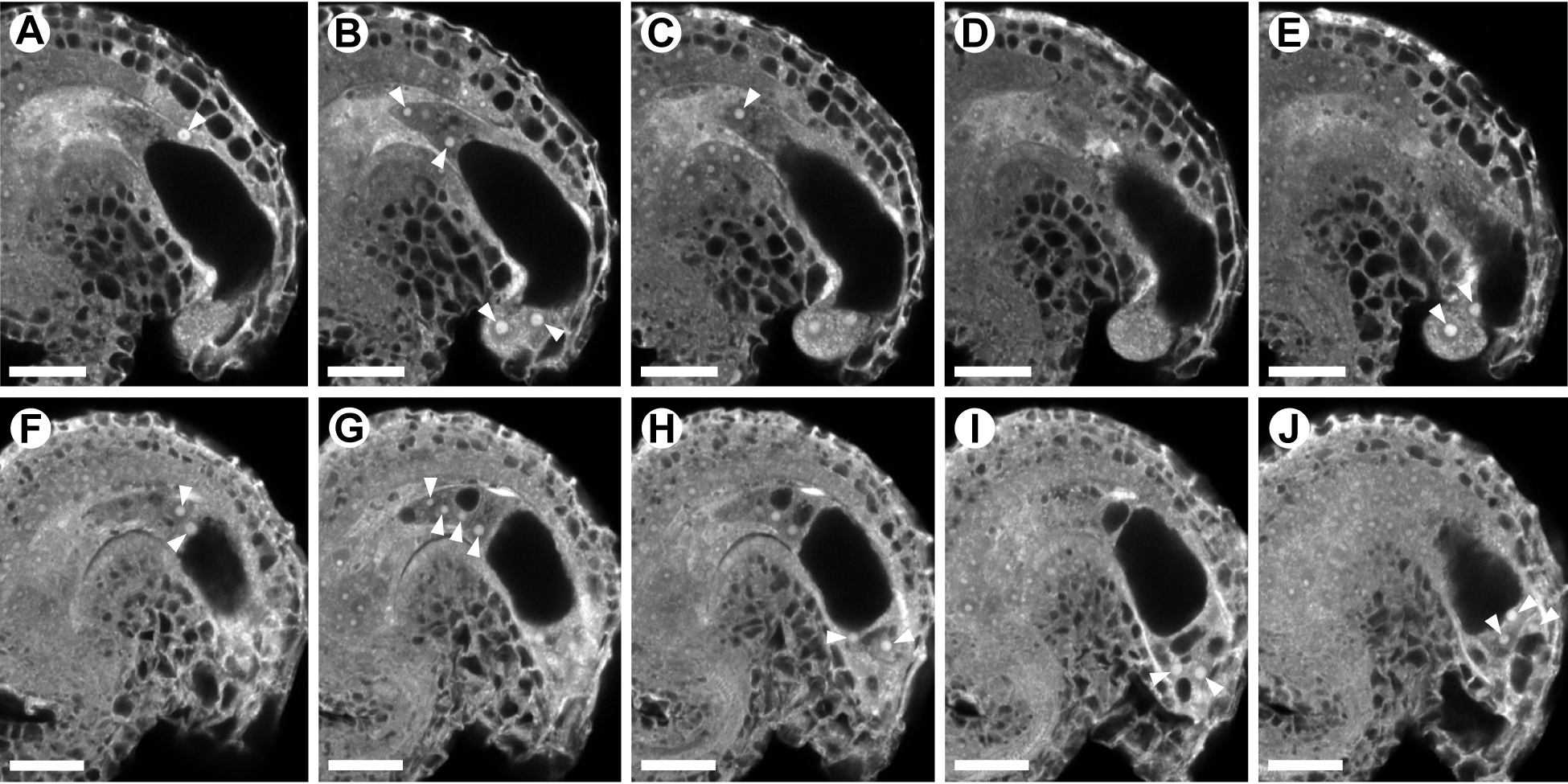

Supplement: Figure S5 — Additional Z-stack series of myb64 myb119 gametophytes. (A–E) A Z-stack series of the coenocytic, eight-nucleate myb64 myb119 female gametophyte depicted in Figure 3E. Eight nuclei are indicated by arrowheads. (F–J) A Z-stack series of the cellularized myb64 myb119 female gametophyte depicted in Figure 3G. Thirteen nuclei are indicated by arrowheads. Scale bars are 20 µm. (TIF) [file pgen.1003783.s005.tif]

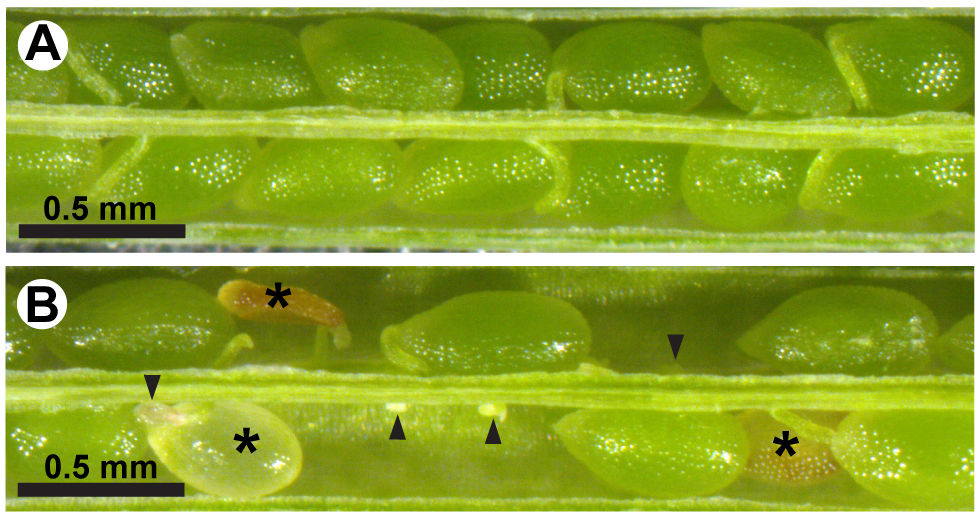

Supplement: Figure S6 — Silique phenotype of myb64 myb119 plants. (A and B) Opened siliques from a self-fertilized wild-type plant (A) and a self-fertilized myb64-1/MYB64 myb119-3/myb119-3 plant (B). Arrowheads indicate desiccated ovules and asterisks indicate white or collapsed seed-like structures. (TIF) [file pgen.1003783.s006.tif]

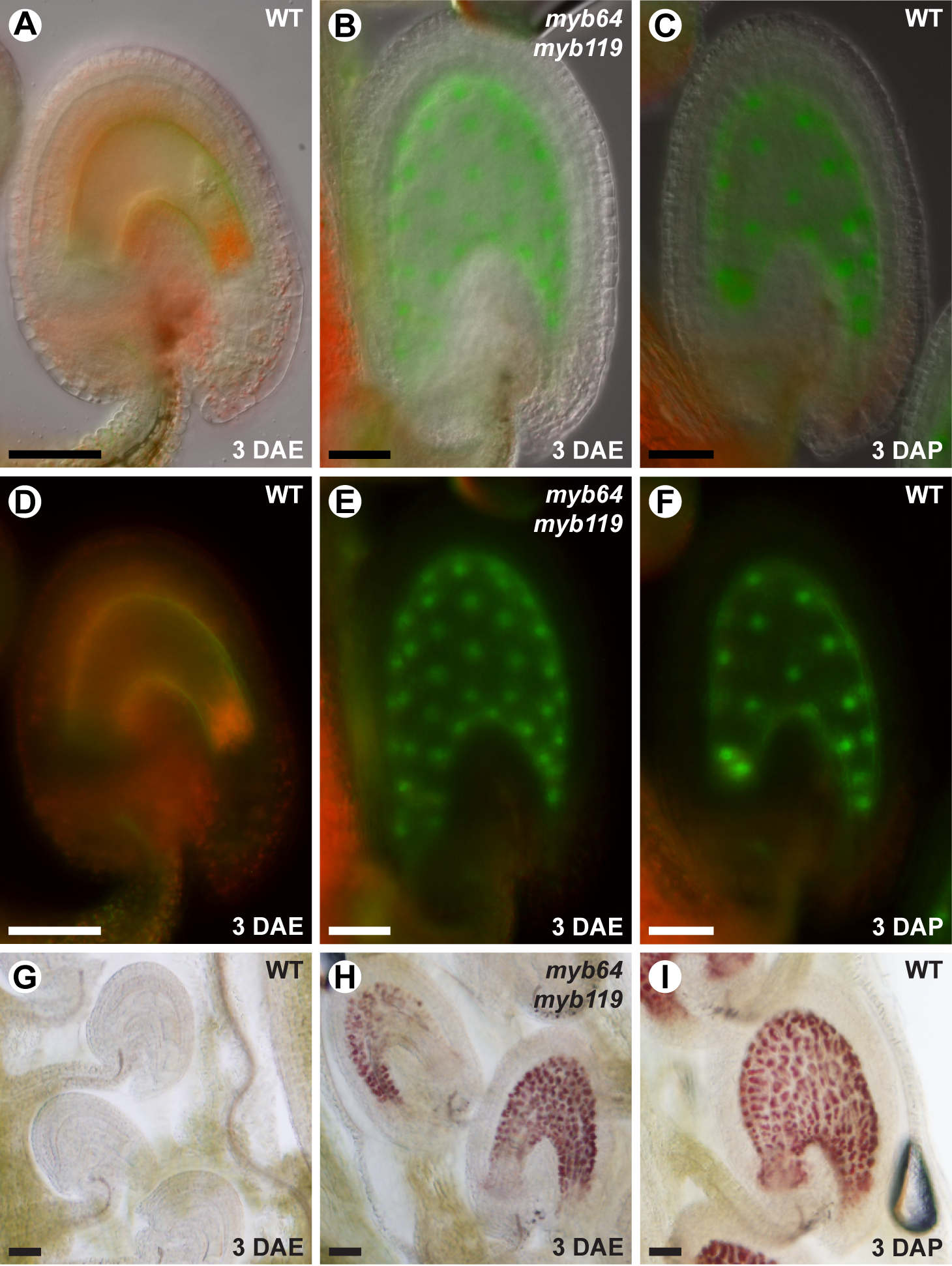

Supplement: Figure S7 — Autonomous endosperm and seed coat development in myb64 myb119 gametophytes. (A–F) Analysis of ProAGL62:AGL62-GFP expression in wild-type and myb64 myb119 plants. (A and D) Expression in wild-type seeds at 3 days after emasculation. AGL62-GFP is not detected (N = 257). (B and E) Expression in myb64 myb119 autonomous seed at 3 days after emasculation. AGL62-GFP is detected in the proliferating nuclei. AGL62-GFP expression was observed in 6% (+/−1.7%) of myb64-1/MYB64 myb119-3/myb119-3 ovules at 3 days after emasculation (N = 353). (C and F) Expression in wild-type seeds at 3 days after pollination. AGL62-GFP is detected in proliferating endosperm. (G–I) Vanillin staining of proanthocyanidin accumulation. Proanthocyanidins accumulate in the endothelium layer of the seed coat post-fertilization, and stain dark red in the presence of vanillin in low pH [38], [39]. (G) Wild-type ovules at 3 days after emasculation. Ovules at this stage do not stain positive for proanthocyanidins (N = 351). (H) myb64-4/myb64-4 myb119-1/myb119-1 ovules at 3 days after emasculation. Staining was observed in 49% (+/−9.4%) of myb64-4/myb64-4 myb119-1/myb119-1 ovules (N = 699). (I) A wild-type seed at 3 days after pollination. DAE, days after emasculation; DAP, days after pollination; WT, wild-type. Scale bars are 40 µm. (TIF) [file pgen.1003783.s007.tif]

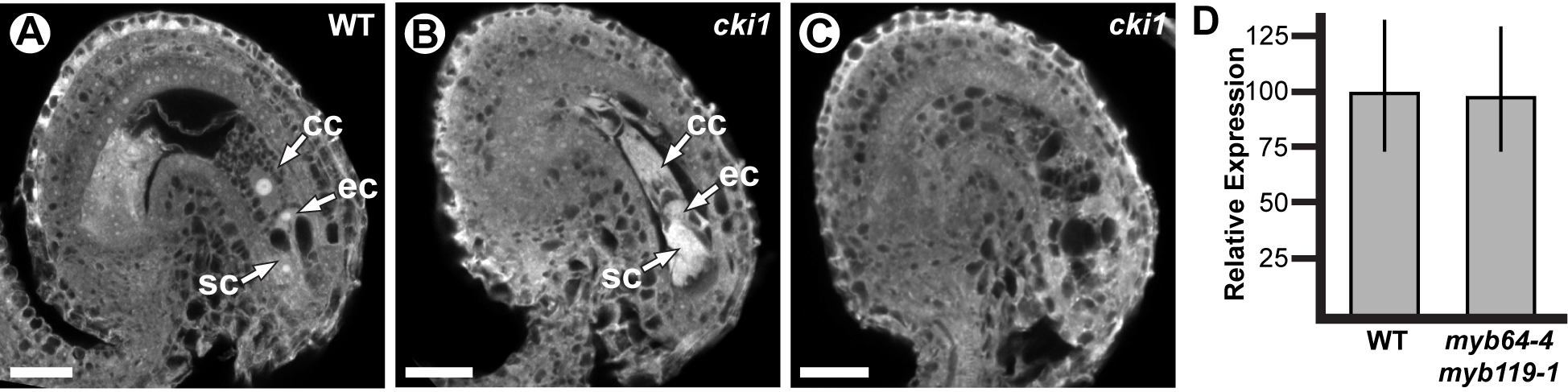

Supplement: Figure S8 — Analysis of the cki1-9 allele and CKI1 expression in myb64 myb119 gametophytes. (A–C) CSLM micrographs of wild-type (A), and cki1-9 (B and C) female gametophytes. (B) An example of the typical cki1 female gametophyte phenotype. The central cell and antipodal cells are degenerated, whereas positioning and cellularization of the egg and synergid cells are unaffected. (C) An example of the less frequent multinucleate/multivacuolate cki1 female gametophyte phenotype. (D) Relative expression of CKI1 in wild-type and myb64-4/myb64-4 myb119-1/myb119-1 ovaries as determined by qRT-PCR. Abbreviations: cc, central cell; ec, egg cell; sc, synergid cell. Error bars indicate standard deviations. WT, wild type. Scale bars are 20 µm. (TIF) [file pgen.1003783.s008.tif]

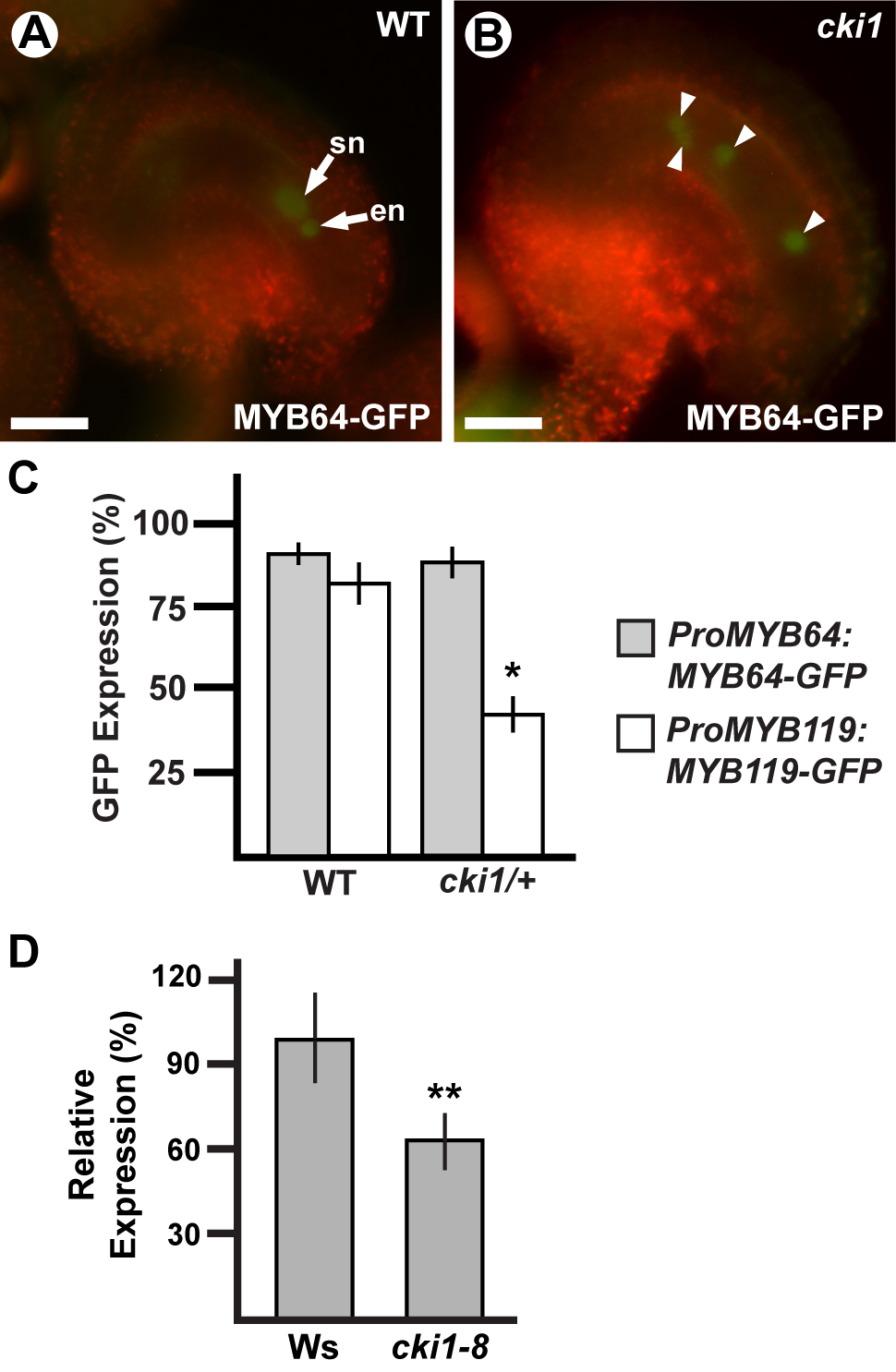

Supplement: Figure S9 — Analysis of MYB64 and MYB119 expression in cki1 female gametophytes. (A and B) Epifluorescent micrographs of ProMYB64:MYB64-GFP expression in wild-type (A) and cki1-9 (B) female gametophytes. Indeterminate nuclei are indicated by arrowheads. (C) Percentage of female gametophytes expressing ProMYB64:MYB64-GFP and ProMYB119:MYB119-GFP in wild type (N = 483 and 608, respectively) and cki1-9/CKI1 (N = 301 and 407, respectively). *, p-val = 3.1E-12 for a two sample t-test as compared to wild type. (D) Relative expression of MYB119 in wild-type and cki1-8/cki1-8 ovaries as determined by qRT-PCR. The average of three biological and three technical replicates normalized to ACTIN2 is reported. We observed a moderate, but significant reduction of MYB119 expression in cki1-8 ovaries. Our ability to detect MYB119 downregulation in this experiment was limited by sporophytic expression of MYB119 in ovaries (Figure S1B), and may reflect differences in accessions (cki1-9 is in the Col-0 background, while cki1-8 is in the Ws background). **, p-val = 6.7E-4 for a two sample t-test as compared to wild type. sn, secondary nucleus of the central cell; en, egg cell nucleus; WT, wild type. (TIF) [file pgen.1003783.s009.tif]
